# How Do Humans Process Audiovisual Cues for Task‐Switching While Walking? An EEG/ERP Study

**DOI:** 10.1111/psyp.70122

**Published:** 2025-08-04

**Authors:** Julian Elias Reiser, Gerhard Rinkenauer, Stefan Arnau, Lewis L. Chuang, Edmund Wascher

**Affiliations:** ^1^ Ergonomics Department Leibniz Research Centre for Working Environment and Human Factors Dortmund Germany; ^2^ Department of Human and Technology, Faculty of Humanities Institute for Media Research, Chemnitz University of Technology Chemnitz Germany

## Abstract

Contemporary work environments require humans to process audiovisual information displays during active locomotion. The attentional demands associated with using devices such as in‐ear headphones and head‐mounted displays may be significantly influenced by varying locomotor demands, yet this relationship remains poorly understood. This study investigates the interplay of information presentation modality, movement state, and cognitive task difficulty. In a virtual reality laboratory, 22 participants performed a cued task‐switch paradigm with three cognitive task difficulty levels while standing, walking, or walking with perturbations on a treadmill. We used a questionnaire, behavioral, and mobile EEG data to investigate cognitive‐motor interference. We find that locomotion interfered with cognitive task performance, and that the presentation modality of task‐switch notification modified the nature of this interference. While auditory cue presentation resulted in faster responses under low cognitive load conditions, visual information presentation was less impaired by higher cognitive and locomotor demands. A detailed analysis of the EEG response to cues addressed these differences in terms of multi‐modal attentional mechanisms. Hence, information presentation on wearable devices should be tailored to the specific task demands, particularly for cognitively demanding information in mobile work settings.

## Abstract 2

This original study is the first to investigate the impact of both auditory and visual information presentation on a cognitive‐motor interference task. With mobile EEG measurements, we uncovered differences in CNV and P3 amplitudes that suggest how differences in modality‐specific attentional processes can result in different patterns of cognitive‐motor interference. Our results inform the design of notification displays in mobile work environments, so that complex cognitive task information should be displayed visually while walking.

## 1 Introduction

Information technology has impacted how we perform everyday tasks, even in traditional domains that involve manual labor (Fernández‐Caramés 2024; Mark et al. 2021). Wearable audiovisual displays, such as head‐mounted displays (HMDs) and in‐ear headphones, increasingly deliver task‐relevant information in mobile settings (Grosse et al. 2017; Guo et al. 2015)—often during active locomotion. Compared to traditional information displays (e.g., paper‐based instructions), wearable displays decrease the time of information uptake and increase productivity (Wu et al. 2016). However, the impact of real‐time information on cognitive workload remains uncertain. A critical challenge in such scenarios is cognitive‐motor interference (CMI), where concurrent cognitive and motor tasks compete for shared attentional resources, often degrading performance (Bloem et al. 2001; Lajoie et al. 1993). Most often, though, decrements manifest in the cognitive task, as postural stability is prioritized to prevent injury (posture‐first hypothesis) (Shumway‐Cook et al. 1997), also in young and healthy populations (Bloem et al. 2001; Lajoie et al. 1993; Yogev‐Seligmann et al. 2008). Theories of CMI often attribute dual‐task costs to bottlenecks in executive functions mediated by frontal–parietal networks (for a review see Leone et al. 2017), though no single neural locus explains all these effects (De Jong 1993). This suggests that executive functions are strongly involved in CMI processes, as these are attributed to be managed in frontal cortical regions.

Critically, the modality of information presentation (visual vs. auditory) may modulate CMI by engaging different sensory pathways. Wickens' (2002) multiple resource theory postulates that visual cues compete with locomotion for visuospatial resources, whereas auditory cues avoid this overlap. However, empirical findings highlight conflicting evidence: while some studies report advantages for visual cues during high cognitive load, others show auditory cues reduce interference in simpler tasks (Kreutzfeldt et al. 2019a, 2019b). The effect of CMI can also be aggregated by higher locomotor effort—like having to avoid obstacles during locomotion (Sampson 1993). Mobile EEG studies further complicate this picture, as it was shown that locomotion can have detrimental effects on visual task execution behavior—especially when the cognitive task is challenging (Shaw et al. 2018)—as well as on auditory cognitive tasks (De Vos et al. 2014; Reiser et al. 2019, 2021). This might be explained by the differing cognitive transformations that visual and auditory cue information must undergo. While visual cue information can be stored both in the visual–spatial sketchpad and in the auditory loop (in the form of inner speech), auditory cue information might need to be transformed into a visual representation after encoding to be able to correctly answer a visual target stimulus information (Baddeley et al. 2001; Emerson and Miyake 2003).

The present study addresses two unresolved questions: (1) How do visual and auditory cue modalities differentially affect attentional resource allocation during CMI? (2) Do these effects vary with motor difficulty (e.g., perturbed walking) and cognitive task complexity (e.g., task‐switching)? To investigate this, we employed a cued task‐switching paradigm where participants categorized visual targets after receiving modality‐specific cues (HMD or headphones) while standing, walking, or navigating treadmill perturbations. This design isolates two processing stages:

1. Cue‐driven rule retrieval and preparation: After cue presentation, participants engage in task‐set reconfiguration, retrieving rules and preparing motor responses. This stage is indexed by the contingent negative variation (CNV), a slow cortical potential maximal at fronto‐central sites. The CNV reflects anticipatory attention and motor preparation, showing higher amplitudes when expected task demands and cognitive load increase (Brunia and van Boxtel 2001; Nicholson et al. 2005; Swainson et al. 2003; Walter et al. 1964). In task‐switching paradigms, switch trials typically elicit stronger CNV amplitudes than repeat trials due to the need to suppress previous task rules (Jost et al. 2008; Kreutzfeldt et al. 2017). Although most of the results are based on experiments with seated participants, a mobile EEG study on auditory task‐switching demonstrated a significant negative impact of task load on CNV amplitudes, with a marginal interaction with walking difficulty. Here, lower amplitudes were found during walking compared to standing in an outside‐the‐lab environment (Reiser et al. 2021).
2. Target processing and response execution: Once the target appears, attentional resources are allocated to stimulus categorization and response selection. This stage is captured by the P3 component, a centro‐parietal positivity peaking 300–600 ms post‐stimulus. Previous studies have found that the P3 component is a correlate of cognitive resource deployment in dual‐task paradigms (Kok 2001). In many CMI studies, the amplitude of the P3, time‐locked to a cognitive task stimulus, decreases with increasing motor loads. If locomotor loads were kept the same and the cognitive task varied in difficulty, P3 amplitudes were shown to increase with increasing cognitive task demands (Bradford et al. 2019; Ladouce et al. 2019; Malcolm et al. 2018; Protzak et al. 2021; Reiser et al. 2022).

To comprehensively evaluate cognitive‐motor interference, we assessed subjective workload via questionnaires and quantified locomotor stability through gait metrics, alongside EEG and behavioral measures. We hypothesized that:

1. *Questionnaire data*: Perceived workload will increase with motor and cognitive difficulty. Visual cues will elevate workload due to visuospatial competition (Wickens 2002), while auditory cues may impose additional demands from cross‐modality transformations.
2. *Response times/accuracy*: Increasing motor difficulty (e.g., perturbed walking) and cognitive demands will slow responses and reduce accuracy. Auditory cues will exacerbate interference in complex tasks, whereas visual cues will impair performance in simpler tasks.
3. *Gait metrics*: Stride times will shorten during perturbed walking to stabilize posture but might lengthen in mixed‐task blocks due to dual‐task costs.
4. *Contingent negative variation* (CNV): Fronto‐central CNV amplitudes will decrease with motor difficulty but increase with cognitive demands (e.g., switch > repeat trials). Visual cues will show greater attenuation during locomotion due to visuospatial resource competition.
5. *P3 component*: Centro‐parietal P3 amplitudes, indexing target processing, will diminish with motor load but amplify with cognitive complexity. Auditory cues may mitigate P3 suppression by reducing visuospatial overlap but impose transformation costs.

By integrating mobile EEG, behavioral metrics, and subjective workload assessments, this work clarifies how wearable displays influence cognitive‐motor interactions in ecologically valid settings. Our findings inform the design of adaptive interfaces that balance modality‐specific benefits against the cognitive costs of cross‐modality processing.

## 2 Method

### 2.1 Participants

Twenty‐two healthy participants (9 male, 13 female) were recruited by online advertisements in 2021. All participants had normal or corrected‐to‐normal vision, no hearing impairments, were right‐handed, and had no motor or gait impairments. Participants were without any known prior or present neurologic or psychiatric condition. The sample's age ranged from 20 to 30 years (*M* = 24.27, SE = 2.96). Subjects either received course credit or €10 per hour as compensation and gave their informed written consent. The study was approved by the local ethics committee of the Leibniz Research Centre for Working Environment and Human Factors and was conducted in accordance with the Declaration of Helsinki.

### 2.2 Apparatus and Stimuli

**FIGURE 1:**
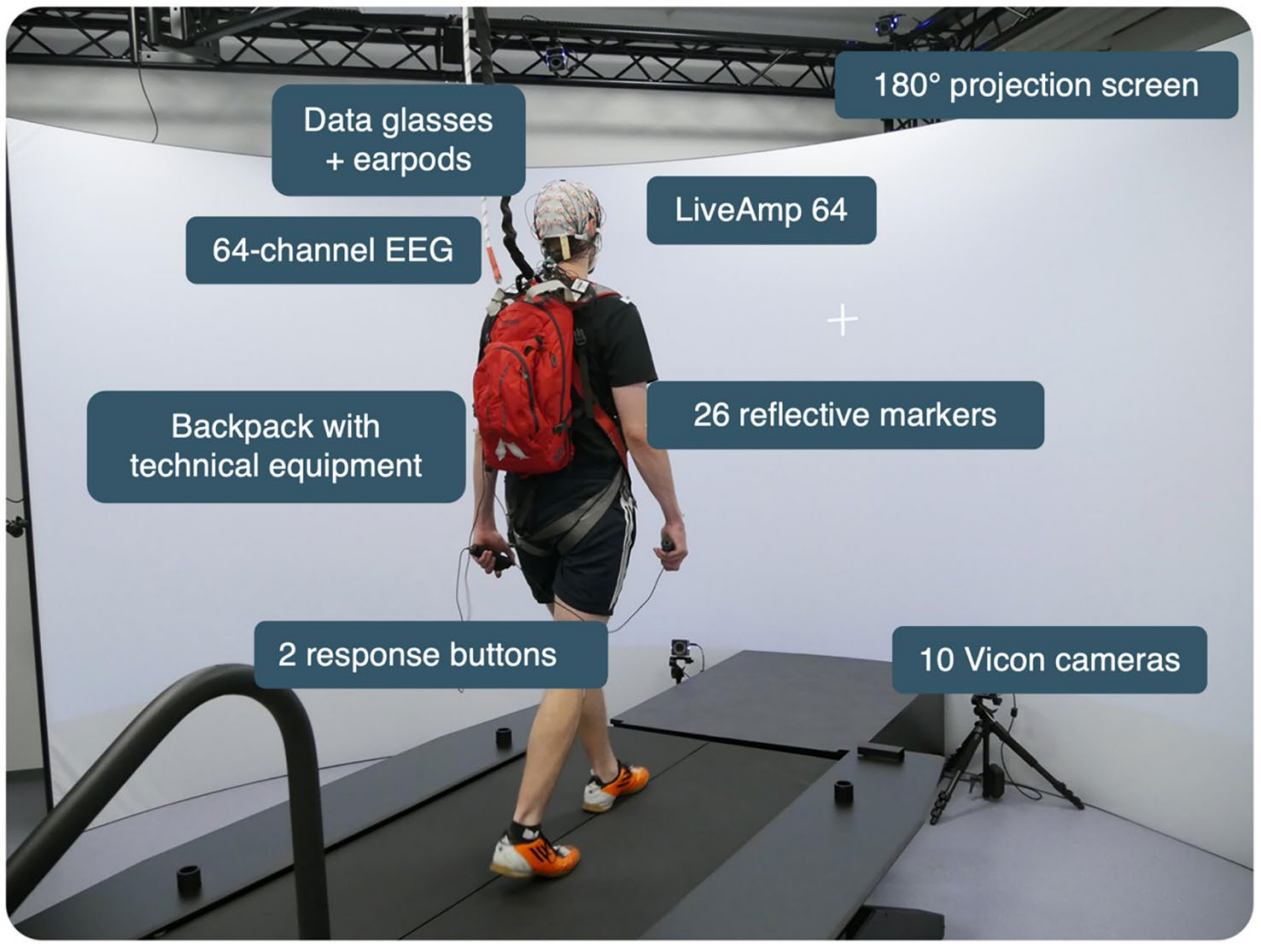
GRAIL laboratory at the Leibniz Research Centre for Working Environment and Human Factors.

All parts of the experiments were performed in the institute's Gait Real‐time Analysis Interactive Laboratory (GRAIL, Motek, NL, see Figure 1). We used a dual‐belt treadmill with tilt and sway functionality, a 10‐camera infrared‐based motion capture system (Vicon Motion Systems, Oxford, UK), and three projectors (WUX450ST, Canon, Japan) projecting onto a 180° curved projection screen (circular screen 5 m diameter, 2.9 m height). DFlow (version 2) software was used to control the treadmill and the projections.

The mobile data collection set‐up consisted of a 64‐electrode cap (ActiCap Snap; Brain Products, Gilching, Germany), two connected mobile amplifiers (LiveAmp; Brain Products, Gilching, Germany), and a backpack containing the amplifiers' trigger extension to allow for manual responses (LiveAmp Sensor and Trigger Extension; Brain Products, Gilching, Germany). In this backpack, participants also carried a Raspberry Pi (Raspberry Pi Foundation, UK) for visual and auditory stimulation. Participants were prepared with 26 reflective markers at distinct body sites to enable whole‐body motion tracking. They also wore an AiRScouter WD360B (Brother, Japan) HMD with a semi‐transparent screen that was connected to the Raspberry Pi to present monocular visual stimuli. The HMD's translucent screen was placed in front of the left eye.

### 2.3 Procedure

After providing informed consent, participants completed a computerized training session to learn the task rules. The training consisted of three blocks: first, an auditory cue practice block of 20 trials where participants practiced with only one cue type (“red” or “green”) presented auditorily; second, a visual cue practice block of 20 trials with the same practice using visual cue presentation only; and third, a task‐switching practice block of 80 trials with mixed presentation of both cues in semi‐random order. The task required participants to respond to flanker stimuli consisting of 5‐letter strings such as “SSSSS” or “SSHSS” by pressing left or right buttons based on the central letter. Response mapping was determined by the cue color, where, for example, “green” indicated the left button for the target “H” and right button for the target “S”, while “red” indicated the opposite mapping. Presented stimuli were 50% congruent, where the central letter matched flankers, and 50% incongruent. Participants repeated training blocks until achieving at least 75% accuracy. Also, within mixed blocks, the number of repeat and switch trials was balanced.

The experiment consisted of six conditions combining three motor states (standing, walking, and perturbed walking) with two cue modalities (auditory and visual). Each condition included two consecutive blocks: first, a repeat‐only sub‐block of 80 trials with the same cue throughout to establish baseline performance, followed by a mixed‐task sub‐block of 160 trials containing 50% repeat trials and 50% switch trials with pseudo‐random cue alternation. Cue modalities alternated between conditions, with order counterbalanced across participants. The total experiment duration was approximately 4 h, including breaks.

Following successful training, participants were fitted with a 64‐electrode EEG cap with impedances maintained below 10 kΩ, 26 motion‐tracking markers at prominent body locations, and a safety harness. They wore a backpack containing the trigger box, Raspberry Pi, HMD control box, and power banks. The entire preparation process took approximately 60 min.

Before the main experiment, participants completed three 2‐min baseline recordings: standing still, walking at self‐paced speed determined through stepwise adjustments, and walking with horizontal treadmill perturbations (max. perturbation speed = 0.11 m/s, max. acceleration = 0.65 m/s^2^, overall duration of 800 ms from displacement onset to a return to normal). In later condition blocks, when the cognitive task was performed simultaneously, a single perturbation could occur at any point during each trial. To prevent participants from anticipating perturbations, 10% of trials were left unperturbed as catch‐trials.

**FIGURE 2:**
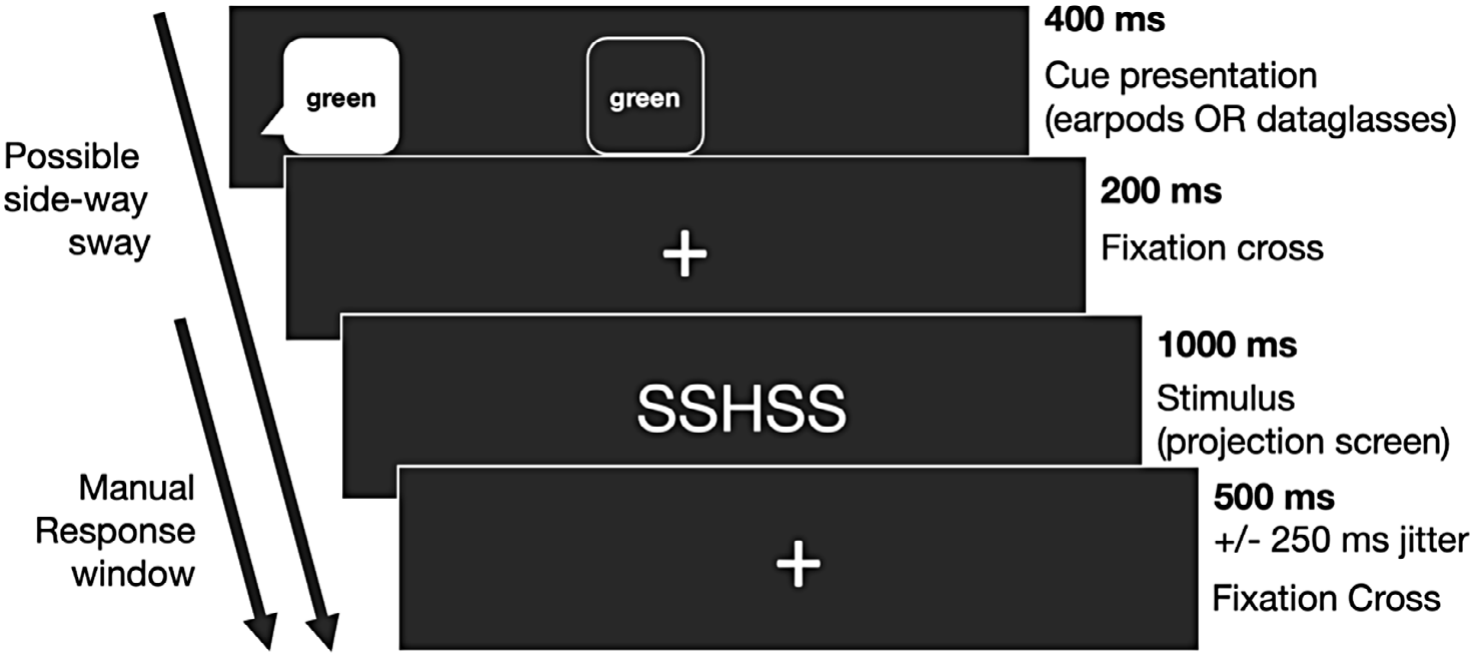
The course of a trial with a mean duration of 2100 ms. A trial started with the presentation of a cue stimulus either auditorily via headphones or visually via an HMD. The modality of cue presentation was alternated in a block‐wise manner. The cue stimulus was followed by a fixation cross for 200 ms and the target stimulus presentation on the projection screen in front of the participant for 1000 ms. The target stimulus was followed by another fixation cross presentation for 500 ms with a jitter of ±250 ms.

The main experiment used a cued task‐switching paradigm illustrated in Figure 2. Each trial followed a fixed sequence, beginning with cue presentation for 400 ms, delivered either auditorily via headphones or visually via HMD, followed by a fixation cross displayed on the projection screen for 200 ms, then the target flanker array on the projection screen for 1000 ms, and finally a response window of 500 ms with ±250 ms jitter during which participants responded via button press.

### 2.4 Questionnaire and Behavioral Data

To assess subjective workload, a German translation of the NASA Task‐Load indeX (NASA‐TLX; Hart and Staveland 1988) was used. Participants were required to complete the questionnaire after the completion of each task. The unweighted mean score over all sub‐dimensions was analyzed. Response times were calculated as the latency between target stimulus presentation and manual response in ms. Accuracy was calculated as the ratio of correct responses compared to the number of all trials in a condition block.

GRAIL motion capturing data was used to calculate step events that were added to the EEG event structure using custom Matlab scripts. A left or right step was detected when the 0.5–5 Hz band‐pass filtered left or right heel marker time‐series indicated a turning point on the vertical axis. Mean stride time was calculated as the mean time difference between two consecutive left steps in ms per condition. Stride time variance was calculated as the mean variance between two consecutive left steps.

### 2.5 Electrophysiological Data Acquisition

EEG was recorded using 64 electrodes in a standard 10–20‐system montage (AF7, AF3, AF4, AF8, Fp1, Fp2, F7, F5, F3, F1, Fz, F2, F4, F6, F8, FC5, FC3, FC1, FC2, FC4, FC6, FT9, FT7, T7, C5, C3, C1, Cz, C2, C4, C6, T8, FT8, FT10, TP9, TP7, CP5, CP3, CP1, CPz, CP2, CP4, CP6, TP8, TP10, P7, P5, P3, P1, Pz, P2, P4, P6, P8, PO9, PO7, PO3, POz, PO4, PO8, PO10, O1, Oz, O2). FCz was used as the online reference electrode, and AFz as the ground electrode. After fitting a tight cap with a circumference ranging from 54 to 60 cm over the participant's head, actively shielded electrodes were inserted into the embedded electrode holders and filled with conductive gel until impedances reached 10 kΩ or less. It was ensured that cables did not cross as they were routed along the head using the cap's loopholes before they were routed into the LiveAmp 64. Data were recorded directly to a micro‐SD card inserted into the amplifier at a sampling rate of 500 Hz and a bit depth of 24 bits. At the same time, the online data stream which was received from the amplifier's Bluetooth connection was monitored on a laptop using the Brainvision Recorder software (Brain Products GmbH). At the end of the experiment, the EEG data were transferred from the SD card to the laptop using the LiveAmp File Converter software (Brain Products GmbH).

### 2.6 Data Processing

In the first step, excess triggers that were recorded by accident (e.g., by starting a wrong condition) were manually removed from the datasets. Then, all data between condition blocks were excluded, and the data were high‐pass filtered at 0.1 Hz using a second‐order Butterworth filter; both functions were taken from the ERPLAB toolbox (Lopez‐Calderon and Luck 2014). Next, the GRAIL data was merged with the EEG dataset.

After the data merging step, we followed the bemobil prep pipeline (Klug, Jeung, et al. 2022) to further clean the data. First, 50 Hz line noise was removed using the zapline function (de Cheveigné 2020), then data channels with poor quality were identified, rejected, and interpolated (*M* = 5.68, SD = 3.81). From here, a separate ICA dataset was created as a duplicate of our EEG data that was high‐pass filtered at 1.5 Hz (filter order 1651). Before adaptive mixture independent component analysis (AMICA) calculations, the data rank was reduced using a PCA (rank reduction: 64 [overall electrodes] − 1 [average reference] − total number of rejected channels). This dataset was then used to compute an AMICA with a likelihood‐based auto‐rejection procedure (Klug, Berg, and Gramann 2022) to clean data from artifactual or unlikely data points (10 likelihood‐based rejection iterations before AMICA, maximum iterations of the AMICA = 2000, AMICA auto‐rejection enabled). After AMICA, dipoles were fitted for each IC to obtain optimal results from ICLabel (Version 1.6) classification (Pion‐Tonachini et al. 2019). Using the results from ICLabel, we rejected all independent components that were classified as reflecting brain activity with less than 50% probability. Due to the high brain class threshold, 63% (SD = 10.35, *N* = 38.27) of ICs were excluded before epoching. Before averaging within subjects, subject‐wise continuous data (pop_rejcont with elecrange = all eegchans, freqlimit = [20 40], threshold = 10, epochlength = 0.5, contiguous = 4, addlength = 0.25, taper = hamming) and subject‐wise epoch rejection was performed (pop_autorej with threshold = 500, startprob = 5, maxrej = 10). On average, 22% of trials were rejected (SD = 8.30).

### 2.7 EEG Parametrization and Analysis

#### 2.7.1 ERPs

The preprocessed data were averaged for each channel and each experimental condition. Within these conditions, the data were baselined from −800 to −600 ms prior to target onset (right before the onset of the cue) for CNV analysis. For target‐related P3 analysis, the time window ranging from −200 to 0 ms relative to target onset was used as the baseline period. To quantify amplitudes and latencies of the ERP components of interest, we used the factorial mass univariate analysis (FMUA) approach presented by Fields and Kuperberg (2020). FMUA is used to compute tests on all electrodes and time points in a time window of interest and controls for family‐wise errors. We used this approach to compute FMUAs with the factors cognitive difficulty (repeat‐only, mixed‐repeat, mixed‐switch trial) and motor difficulty (stand, walk, perturbation) in a priori defined time windows and regions of interest (see below). A significance level of 0.05 was used for all tests. The computed ANOVAs were corrected for multiple comparisons with the Permutation Based Cluster Mass technique (Groppe et al. 2011). Here, data points that are spatially and temporally adjacent are clustered if they exceed the cluster inclusion threshold. Then, all *F*‐values in a cluster are summed and compared to a null distribution created by performing 10,000 permutations. This cluster‐based technique is useful to uncover ERP effects, as clusters are more likely to occur in a significant pattern compared to signal noise (Fields and Kuperberg 2020; Groppe et al. 2011). Follow‐up FMUAs were conducted if a factor had more than two levels or in case of a significant interaction (recommended instead of *t*‐tests when a clustering technique was used before; Fields and Kuperberg 2020). See the authors' GitHub repository for more information (https://github.com/ericcfields/FMUT/wiki/Using‐FMUT). All significant FMUA comparisons' *F*‐value raster figures can be found in the Supporting Information.

#### 2.7.2 Fronto‐Central CNV

We investigated the CNV in a fronto‐central electrode patch (F1, F2, Fz, FC1, FC2, Cz, C1, C2). The CNV was investigated in the time window between −150 ms before and 50 ms after target stimulus onset.

#### 2.7.3 Centro‐Parieto‐Occipital P3

The P3 component was investigated in a parieto‐occipital electrode patch. The P3 component was analyzed in the time window between 300 and 600 ms after target stimulus onset (CPz, CP1, CP2, Pz, P1, P2, P3, P4, POz, PO3, PO4, Oz, O1, O2).

#### 2.7.4 Multiple Cluster Handling

When multiple significant clusters emerged for a single effect, each cluster was treated as a separate statistical entity. For publication purposes, we report the most significant cluster (lowest *p*‐value) per effect in simplified tables, while comprehensive reporting includes all significant clusters (see Supporting Information).

#### 2.7.5 Post Hoc Analyses

For significant main effects and interactions, we conducted cluster‐based post hoc comparisons by extracting mean amplitudes within significant cluster boundaries for each experimental condition. Pairwise comparisons between conditions were performed using *t*‐tests with pooled standard error calculations, and partial eta squared was computed for effect size estimation. For a list of all comparisons, see Supporting Information.

Given the exploratory nature of our study and our use of factorial mass univariate analysis, we deliberately adopted broad time‐windows within the usual range of the used ERP components. This approach maximized our sensitivity to detect any emerging effects across ERP components within this novel experimental paradigm.

### 2.8 Statistical Analysis

The experimental design left us with three possible factors for analyses: locomotion difficulty (standing vs. walking vs. perturbed walking), task difficulty (repeat‐only conditions, repeat trials in a mixed‐task conditions, switch trials in a mixed‐task conditions), and modality (visual cue presentation vs. auditory cue presentation).

Repeated‐measures ANOVAs for subjective, response, and gait data were performed with Matlab's built‐in functions (fitrm, ranova). Post hoc tests were performed using the Tukey–Kramer procedure. When a sphericity assumption was violated, *p*‐values were Greenhouse–Geisser corrected (indicated by *p*_GG_). When necessary, family‐wise errors were corrected for false discovery rate as outlined by Cramer et al. (2016). Whenever used, FDR‐adjusted critical *p*‐values (*p*_crit_) are provided. Effect sizes for rmANOVAs are reported as adjusted partial eta squared as described by Mordkoff (2019).

We analyzed questionnaire data using a 3 (motor task difficulty) × 2 (cognitive task difficulty) × 2 (cue modality) repeated measures ANOVA design. Task difficulty was collapsed into two factor levels (repeat‐only condition vs. mixed task condition) because questionnaires were only administered at the end of a block. Therefore, we could not isolate the individual contributions of repeat and switch trials to the dependent variables within the mixed‐task blocks.

For the gait analysis, only walking conditions were included, resulting in a 2 (motor difficulty) × 2 (cognitive task difficulty) × 2 (cue modality) design.

Regarding response‐related measures (response times and accuracy) we conducted a 3 (motor task difficulty) × 3 (cognitive task difficulty) × 2 (cue modality) repeated measures ANOVA.

For EEG‐related analyses, we split the statistical comparisons into two modality‐specific 3 (motor task difficulty) × 3 (cognitive task difficulty) analyses. The reason for this split was that EEG morphologies showed distinctly different patterns between auditory and visual cue conditions.

## 3 Results

### 3.1 Subjective Workload

#### 3.1.1 Motor Difficulty

A significant main effect was found, *F*(2,42) = 11.52, *p* < 0.001, *η* ^2^ = 0.32, indicating that motor conditions differentially affected perceived workload. Post hoc comparisons revealed that perturbation conditions resulted in significantly higher workload ratings than both standing (*p* = 0.01) and walking (*p* < 0.001). No significant difference was observed between standing and walking conditions (*p* = 0.86).

#### 3.1.2 Cognitive Difficulty

A highly significant main effect emerged, *F*(1,21) = 111.81, *p* < 0.001, *η* ^2^ = 0.83, with substantially higher workload ratings in mixed task blocks compared to repeat‐only blocks.

**FIGURE 3:**
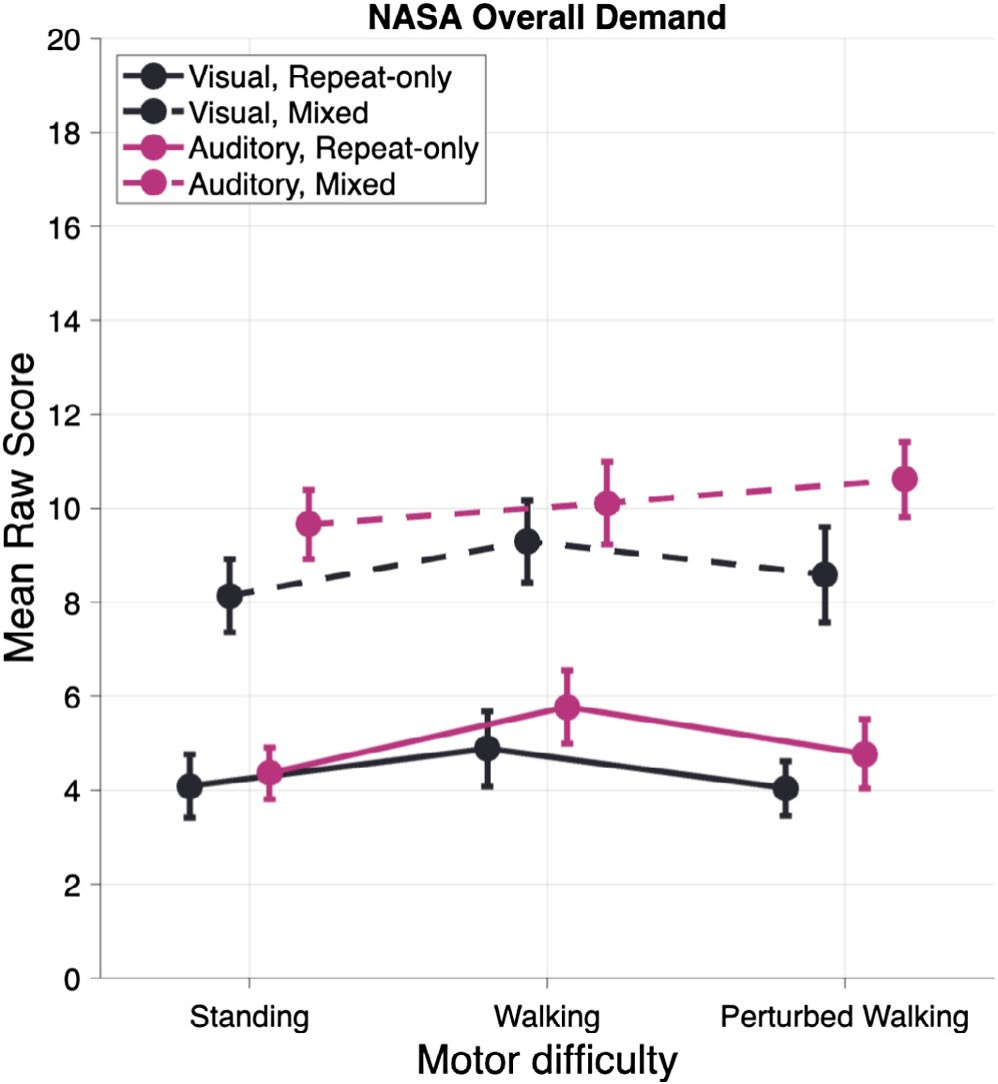
NASA‐TLX mean raw scores. For the calculation of the overall mean scores, all subdimensions (cognitive demands, physical demands, time demands, performance, effort, and frustration) were averaged.

These results indicate that cognitive task complexity was the primary driver of perceived workload, with mixed cognitive demands substantially increasing subjective ratings. Motor perturbations specifically contributed to increased workload beyond regular locomotion, suggesting that balance challenges add a distinct cognitive‐motor burden compared to steady‐state walking or standing. For a detailed overview of ANOVA results, see Table 1 and Figure 3.

**TABLE 1:** Subjective workload repeated measures ANOVA results.

Effect	df	*F*	*p*_GG_	*p*_crit_	Significance	$\eta_{p}^{2}$
Modality	21,1	2.75	0.11	0.03	—	0.07
Motor	42,2	11.52	< 0.001	0.04	*	0.32
Cognitive	21,1	111.81	< 0.001	0.05	*	0.83
Modality × move	42,2	1.03	0.37	0.02	—	0.00
Modality × cognitive	21,1	3.73	0.07	0.04	—	0.11
Move × cognitive	42,2	0.34	0.34	0.01	—	−0.03
Modality × move × cognitive	42,2	0.17	0.79	0.01	—	−0.04

*Note:* Significance of * indicates that the GG‐corrected *p*‐value is smaller than the critical *p*‐value of our FDR‐correction. It does not indicate the level of significance.

### 3.2 Behavior

#### 3.2.1 Gait Analysis

##### 3.2.1.1 Motor Difficulty

Participants showed significantly longer step times during regular treadmill walking (*M* = 1270.90 ms, SD = 105.42) compared to perturbed walking (*M* = 1220.00 ms, SD = 105.33), *F*(1,21) = 45.49, *p* < 0.001, *η* ^2^ = 0.67.

##### 3.2.1.2 Cognitive Difficulty

The main effect of cognitive task difficulty (repeat‐only vs. mixed blocks) was significant, *F*(1,21) = 8.39, *p* = 0.01, *η* ^2^ = 0.25, with mixed blocks resulting in longer stride times (*M* = 1251.4 ms, SD = 109.42) compared to repeat‐only blocks (*M* = 1239.5 ms, SD = 107.10), suggesting an additional cognitive load effect.

##### 3.2.1.3 Summary

These results indicate that walking perturbations decrease step times. Increased cognitive load also affects temporal gait parameters, suggesting that cognitive demands influence locomotor control strategies. For a detailed overview of ANOVA results, see Table 2.

**TABLE 2:** Mean stride time repeated measures ANOVA results.

Effect	df	*F*	*p*_GG_	*p*_crit_	Significance	$\eta_{p}^{2}$
Modality	21,1	0.10	0.76	0.01	—	−0.04
Motor	21,1	45.49	< 0.001	0.05	*	0.67
Cognitive	21,1	8.39	0.01	0.04	*	0.25
Modality × motor	21,1	3.72	0.07	0.04	—	0.11
Modality × cognitive	21,1	1.76	0.20	0.03	—	0.03
Motor × cognitive	21,1	0.34	0.34	0.01	—	−0.01
Modality × motor × cognitive	21,1	0.84	0.37	0.02	—	−0.04

*Note:* Significance of * indicates that the GG‐corrected *p*‐value is smaller than the critical *p*‐value of our FDR‐correction. It does not indicate the level of significance.

#### 3.2.2 Response Times

##### 3.2.2.1 Modality × Cognitive Difficulty

A highly significant interaction was observed, *F*(2,42) = 66.16, *p* < 0.001, *η* ^2^ = 0.75. Post hoc comparisons revealed that response times with auditory modality cues were faster than with visual cues in repeat‐only blocks (*p* < 0.001), while visual modality cues decreased response times in both mixed‐repeat (*p* = 0.003) and mixed‐switch tasks (*p* < 0.001).

##### 3.2.2.2 Motor Difficulty × Cognitive Difficulty

A significant interaction emerged, *F*(4,84) = 4.30, *p* = 0.003, *η* ^2^ = 0.13. Pairwise comparisons showed that perturbation caused faster responses than walking in mixed‐repeat (*p* = 0.015), and perturbation also improved response times compared to both standing (*p* = 0.032) and walking (*p* = 0.001) during mixed‐switch trials. No significant motor difficulty effects were observed in repeat‐only blocks.

##### 3.2.2.3 Main Effects

The significant main effect of modality, *F*(1,21) = 9.69, *p* = 0.005, *η* ^2^ = 0.28, indicated faster overall responses in auditory compared to visual conditions. The main effect of cognitive difficulty, *F*(2,42) = 102.34, *p* < 0.001, *η* ^2^ = 0.82, showed progressive RT increases from repeat‐only to mixed‐repeat to mixed‐switch trials.

##### 3.2.2.4 Summary

These findings demonstrate that both motor and cognitive demands interact with sensory modality, with auditory cueing giving advantages under low cognitive load while visual cueing improves response times under higher cognitive demands.

For a detailed overview of ANOVA results, see Table 3.

**TABLE 3:** Response times repeated measures ANOVA results.

Effect	df	*F*	*p*_GG_	*p*_crit_	Significance	$\eta_{p}^{2}$
Modality	21,1	9.69	0.005	0.04	*	0.28
Motor	42,2	3.18	0.06	0.02	—	0.09
Cognitive	42,2	102.34	< 0.001	0.05	*	0.82
Modality × motor	42,2	1.65	0.20	0.01	—	0.03
Modality × cognitive	42,2	66.16	< 0.001	0.04	*	0.75
Motor × cognitive	84,4	4.30	0.007	0.03	*	0.13
Modality × motor × cognitive	84,4	0.76	0.53	0.01	—	−0.01

*Note:* Significance of * indicates that the GG‐corrected *p*‐value is smaller than the critical *p*‐value of our FDR‐correction. It does not indicate the level of significance.

#### 3.2.3 Response Accuracy

##### 3.2.3.1 Cognitive Difficulty

A highly significant main effect was found, *F*(2,42) = 53.12, *p* < 0.001, *η* ^2^ = 0.70. Post hoc comparisons revealed that accuracy decreased systematically with increasing cognitive difficulty: within repeat‐ony blocks participants showed the highest accuracy, followed by mixed‐repeat (*p* < 0.001), and mixed‐switch trials (*p* < 0.001).

##### 3.2.3.2 Summary

**FIGURE 4:**
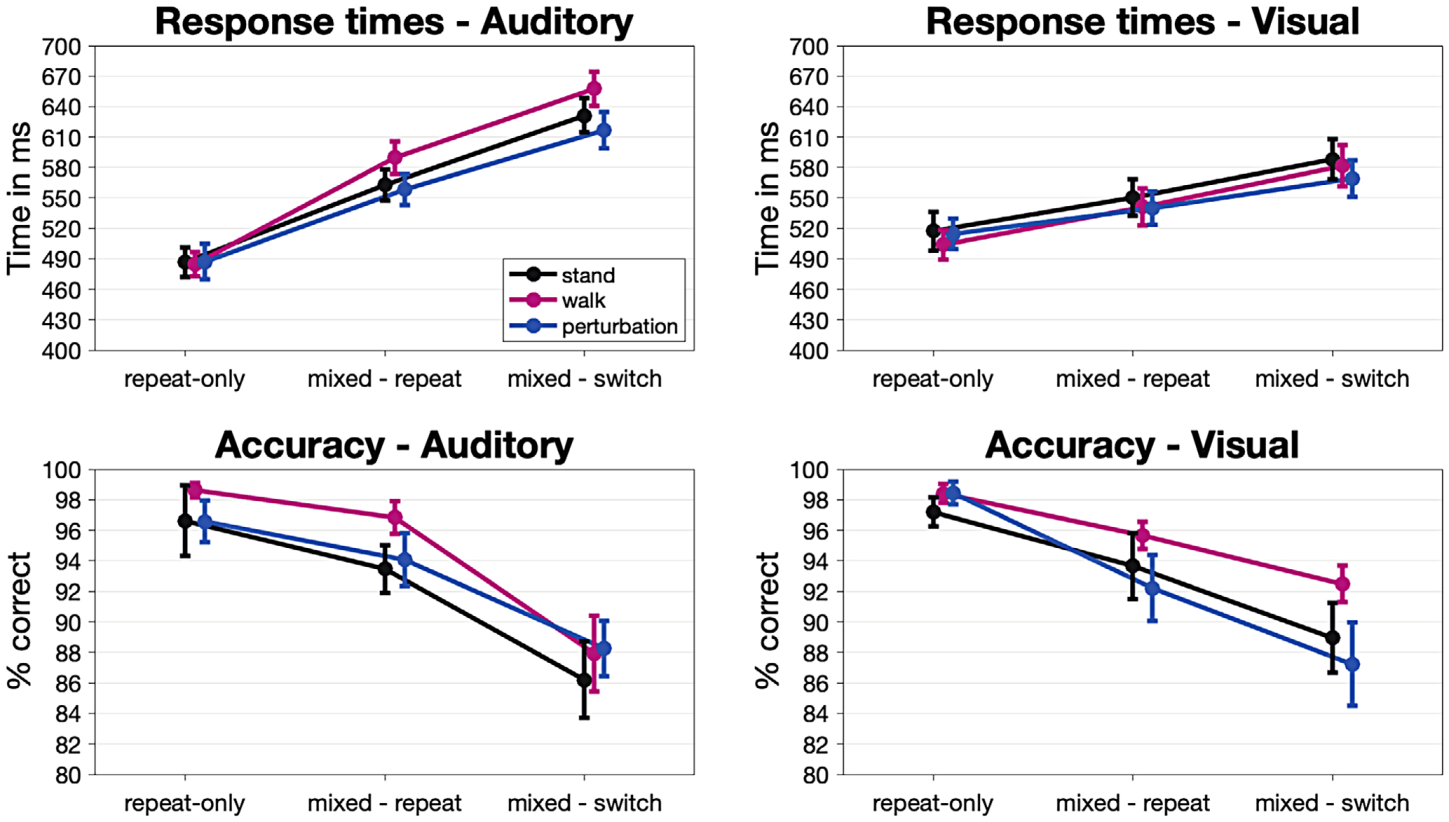
Target stimulus‐related mean response times and accuracies.

These findings demonstrate that cognitive difficulty had a substantial impact on response accuracy, with performance systematically declining as task demands increased. For a detailed overview of ANOVA results, see Table 4. Response times and accuracies are also depicted in Figure 4.

**TABLE 4:** Response accuracy repeated measures ANOVA results.

Effect	df	*F*	*p*_GG_	*p*_crit_	Significance	$\eta_{p}^{2}$
Modality	21,1	0.28	0.60	0.02	—	−0.03
Motor	42,2	2.54	0.10	0.04	—	0.07
Cognitive	42,2	53.12	< 0.001	0.05	*	0.70
Modality × motor	42,2	0.20	0.79	0.01	—	−0.04
Modality × cognitive	42,2	3.30	0.06	0.04	—	0.10
Motor × cognitive	84,4	0.45	0.66	0.01	—	−0.03
Modality × motor × cognitive	84,4	1.43	0.24	0.03	—	0.02

*Note:* Significance of * indicates that the GG‐corrected *p*‐value is smaller than the critical *p*‐value of our FDR‐correction. It does not indicate the level of significance.

### 3.3 ERPs

When looking at ERPs, results from the factorial mass‐univariate analysis will be reported for the fronto‐central CNV and the centro‐parieto‐occipital P3. The analysis was performed on visually presented target stimuli in the reported time windows. A detailed overview of the FMUA ERP results can be found in Table 5.

**TABLE 5:** ERP factorial mass univariate analysis results.

	Modality	Effect	*F*	df	*p*	$\eta_{p}^{2}$	Cluster	Post hoc results
CNV	Auditory	Cognitive	4.18	2,42	0.668	0.166	1	n.s.
		Motor	18.93	2,42	< 0.001	0.474	1	Stand > walk*** (*η* ^2^ = 0.087) Stand = perturbation (*p* = 0.167, *η* ^2^ = 0.015) Walk = perturbation (*p* = 0.060, *η* ^2^ = 0.027)
		Interaction	—	—	—	—	—	n.s.
	Visual	Cognitive	19.67	2,42	< 0.001	0.484	1	Mixed‐switch > repeat‐only** (*η* ^2^ = 0.056) Mixed‐repeat = repeat‐only (*p* = 0.061, *η* ^2^ = 0.027) Mixed‐switch = mixed‐repeat (*p* = 0.491, *η* ^2^ = 0.004)
		Motor	7.76	2,42	0.030	0.270	1	Walk > perturbation** (*η* ^2^ = 0.071) Stand = walk (*p* = 0.127, *η* ^2^ = 0.018) Stand = perturbation (*p* = 0.186, *η* ^2^ = 0.013)
		Interaction	3.37	4,84	0.366	0.138	1	n.s.
P3	Auditory	Cognitive	29.70	2,42	< 0.001	0.586	1	Switch > repeat‐only* (*η* ^2^ = 0.037) Mixed‐repeat = repeat‐only (*p* = 0.295, *η* ^2^ = 0.008)
		Motor	8.96	2,42	0.009	0.299	1	Stand > perturbation* (*η* ^2^ = 0.029) Stand = walk (*p* = 0.145, *η* ^2^ = 0.016)
		Interaction	—	—	0.752	—	—	n.s.
	Visual	Cognitive	30.24	2,42	< 0.001	0.590	1	Repeat‐only > mixed‐repeat*** (*η* ^2^ = 0.167) Repeat‐only > mixed‐switch*** (*η* ^*2*^ = 0.197)
		Motor	12.00	2,42	< 0.001	0.363	2	Stand > walk* (*η* ^2^ = 0.031) Stand > perturbation** (*η* ^2^ = 0.057)
		Interaction	4.11	4,84	0.046	0.164	3	No significant pairwise differences within movement conditions

*Note:* Cluster numbers refer to significant electrode clusters identified in mass‐univariate analysis. Post hoc comparisons show only significant or trending effects with effect sizes.

Abbreviation: $\eta_{p}^{2},\text{partial}\;\mathrm{eta}\;\text{squared}$.

* *p* < 0.05.

** *p* < 0.01.

*** *p* < 0.001.

#### 3.3.1 Fronto‐Central CNV

##### 3.3.1.1 Motor Difficulty

**FIGURE 5:**
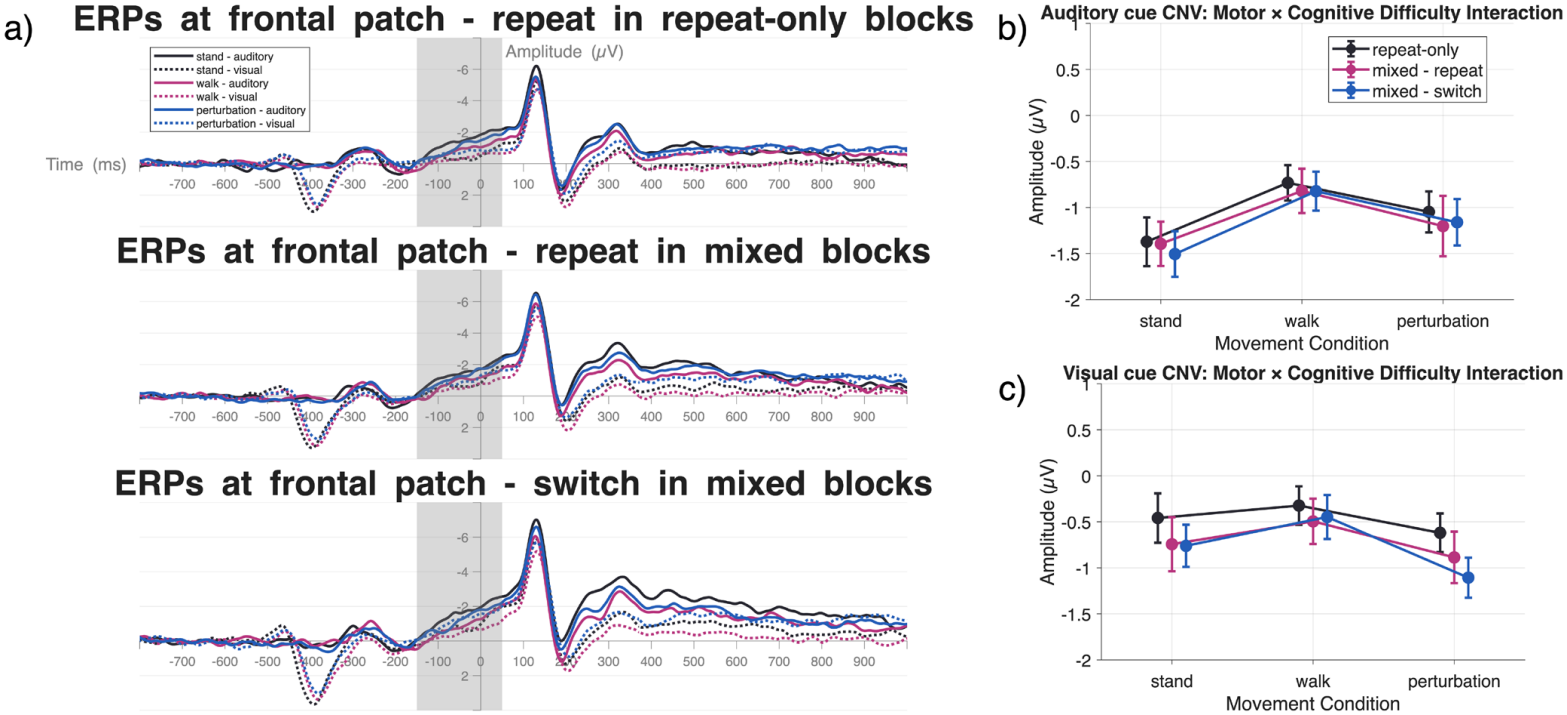
Frontal EEG activity. (a) ERP line plots of mean activation at frontal to central electrodes (F1, F2, Fz, FC1, FC2, Cz, C1, C2). CNV (−150 ms:50 ms) time window are indicated by the gray rectangles. The vertical line at 0 ms represents the presentation of the target stimulus. The cue stimulus was presented at −600 ms. The baseline time window was applied between −800 and −600 ms prior to target stimulus presentation. (b) Mean CNV ERP amplitudes after auditory cue presentation for motor and cognitive difficulty conditions. (c) Mean CNV ERP amplitudes after visual cue presentation for motor and cognitive difficulty conditions. Differences in pre‐target‐stimulus timepoints in (a) originate from the presentation of cue‐stimuli in different modalities (visual and auditory). As the morphologies for these cue‐related ERPs are vastly different, only target‐stimulus‐related ERPs were analyzed. For better comprehensibility, line graphs (b, c) resemble mean activation in the indicated time‐window and might differ from cluster‐based values in the text.

Motor task difficulty significantly modulated CNV amplitudes in both modality conditions. In auditory cue conditions, *F*(2,42) = 18.93, *p* = 0.0001, *η* ^2^ = 0.47, standing elicited the strongest CNV response, followed by walking and perturbed walking. Post hoc comparisons revealed significant differences between standing and walking (*p* < 0.001, *η* ^2^ = 0.09), but not between standing and perturbation (*p* = 0.17, *η* ^2^ = 0.02) or walking and perturbation (*p* = 0.06, *η* ^2^ = 0.03). In visual cue conditions, *F*(2,42) = 7.76, *p* = 0.03, *η* ^2^ = 0.27, perturbed walking showed the strongest CNV, followed by standing and regular walking, with significant differences between regular and perturbed walking (*p* = 0.002, *η* ^2^ = 0.07) Figure 5.

##### 3.3.1.2 Cognitive Difficulty

**FIGURE 6:**
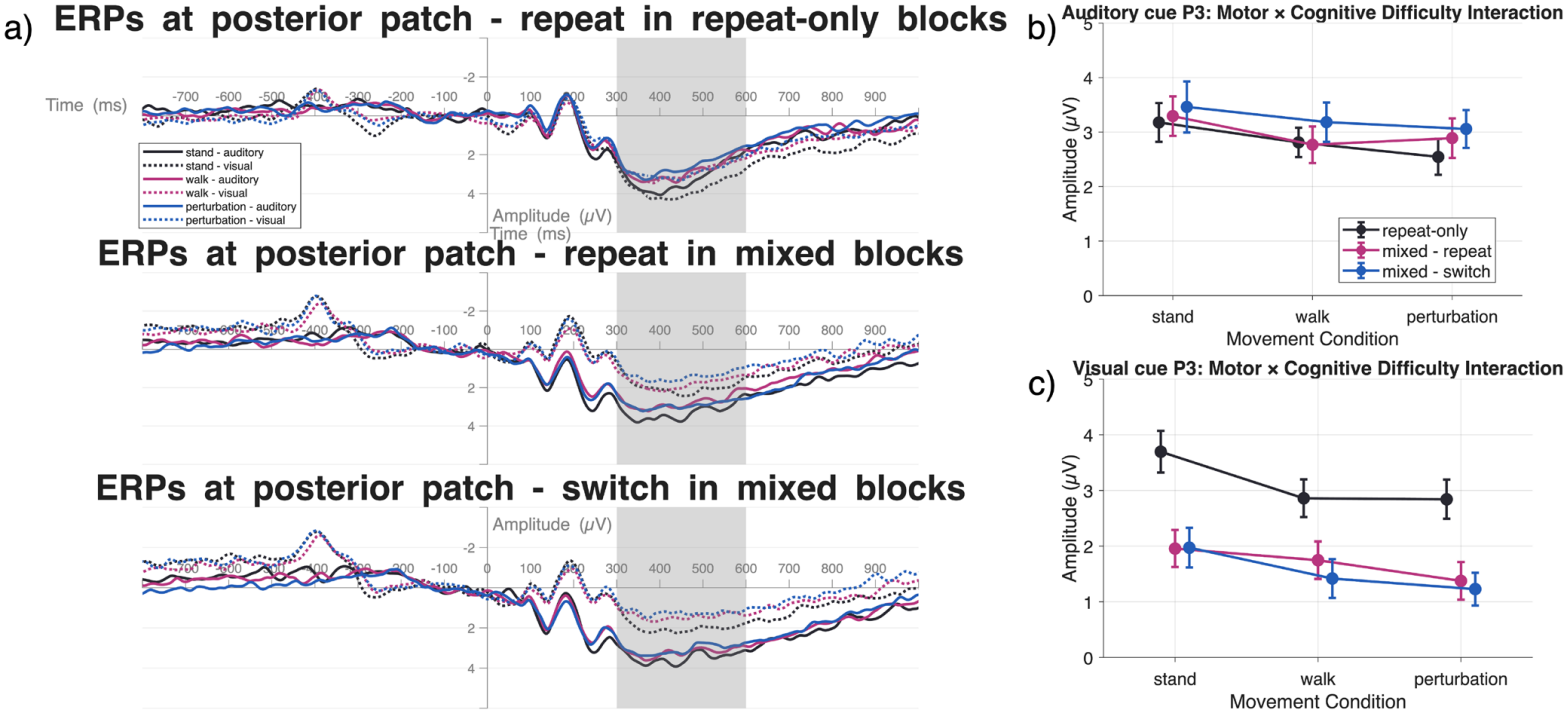
Posterior EEG activity. (a) ERP line plots of mean activation at centro‐parietal to occipital sites (CPz, CP1, CP2, Pz, P1, P2, P3, P4, POz, PO3, PO4, Oz, O1, O2). The P3 (300 ms:600 ms) time window is indicated by the gray rectangles. The vertical line at 0 ms represents the presentation of the target stimulus. The cue stimulus was presented at −600 ms. The baseline time window was applied between −200 and 0 ms prior to the target stimulus presentation. (b) Mean P3 ERP amplitudes after auditory cue presentation for motor and cognitive difficulty conditions. (c) Mean P3 ERP amplitudes after visual cue presentation for motor and cognitive difficulty conditions. Differences in pre‐target‐stimulus timepoints in (a) originate from the presentation of cue‐stimuli in different modalities (visual and auditory). As the morphologies for these cue‐related ERPs are vastly different, only target‐stimulus‐related ERPs were analyzed. For better comprehensibility, line graphs (b, c) resemble mean activation in the indicated time‐window and might differ from cluster‐based values in the text.

Cognitive task difficulty effects differed by modality. No significant effect was found in auditory cue conditions, *F*(2,42) = 4.18, *p* = 0.67, *η* ^2^ = 0.17. However, visual cue conditions showed a highly significant effect, *F*(2,42) = 19.67, *p* = 0.0001, *η* ^2^ = 0.48, with mixed‐switch trials eliciting the strongest CNV response compared to repeat‐only trials (*p* = 0.006, *η* ^2^ = 0.06). Mixed‐repeat trials also differed from repeat‐only trials (*p* = 0.061, *η* ^2^ = 0.03), while mixed‐repeat and mixed‐switch conditions did not differ significantly (*p* = 0.49, *η* ^2^ = 0.00) (Figure 6).

#### 3.3.2 Centro‐Parieto‐Occipital P3

##### 3.3.2.1 Motor Difficulty × Cognitive Difficulty

A significant task difficulty × motor difficulty interaction was found only in visual cue conditions, *F*(4,84) = 4.11, *p* = 0.05, *η* ^2^ = 0.16, while auditory conditions showed no interaction (*p* = 0.75). Post hoc analyses of the visual interaction revealed that the task difficulty effect varied across movement conditions, with the largest differences between repeat‐only and mixed conditions occurring during standing.

The interaction pattern showed distinct profiles across movement conditions. In standing, repeat‐only trials produced the highest P3 amplitudes, followed by mixed‐repeat and mixed‐switch trials. While individual pairwise comparisons within standing did not reach significance (*p*_repeatOnly–mixedRepeat_ = 0.15, *η* ^2^ = 0.05; *p*_repeatOnly–mixedSwitch_ = 0.070, *η* ^2^ = 0.08; *p*_mixedRepeat–mixedSwitch_ = 0.68, *η* ^2^ = 0.00), this condition showed the largest numerical differences between task difficulty levels. During walking, the task difficulty effect was substantially reduced, with repeat‐only, mixed‐repeat, and mixed‐switch trials showing similar amplitudes (all *p* > 0.27). In perturbation conditions, the pattern was intermediate, with repeat‐only trials showing numerically higher amplitudes than mixed‐repeat and mixed‐switch trials, though again without reaching significance in pairwise comparisons (all *p* > 0.15).

##### 3.3.2.2 Motor Difficulty

Motor difficulty significantly affected P3 amplitudes in both modalities. In auditory cue conditions, *F*(2,42) = 8.96, *p* = 0.01, *η* ^2^ = 0.30, standing elicited the highest P3 amplitude, followed by walking and perturbed walking, with significant differences between standing and perturbation (*p* = 0.05, *η* ^2^ = 0.03) but not between standing and walking (*p* = 0.145, *η* ^2^ = 0.01). Visual cue conditions showed a significant movement effect in cluster 2, *F*(2,42) = 11.99, *p* = 0.001, *η* ^2^ = 0.36, where standing produced the highest P3 response, followed by walking and perturbed walking, with significant differences between standing and walking (*p* = 0.04, *η* ^2^ = 0.03) and standing and perturbation (*p* = 0.01, *η* ^2^ = 0.06).

##### 3.3.2.3 Cognitive Difficulty

Cognitive task difficulty showed robust effects across both modalities. In auditory cue conditions, *F*(2,42) = 29.70, *p* = 0.0003, *η* ^2^ = 0.59, switch trials elicited the highest P3 amplitude, followed by repeat and repeat‐only trials. Visual cue conditions showed an even stronger effect, *F*(2,42) = 30.24, *p* = 0.0001, *η* ^2^ = 0.59, with repeat‐only trials showing the highest P3 response, followed by mixed–repeat and mixed–switch trials.

##### 3.3.2.4 Summary

These ERP findings reveal distinct patterns of neural processing across cognitive and motor demands. The CNV component, reflecting motor preparation, was strongly modulated by motor difficulty in both modalities, with standing conditions consistently eliciting the strongest preparatory activity. Task difficulty effects on CNV were modality‐specific, emerging only under visual cue conditions where increased cognitive demands (switch trials) enhanced preparatory processing.

The P3 component showed modality‐specific patterns of cognitive‐motor interaction. In visual cue conditions, the significant interaction indicates that cognitive difficulty effects on P3 amplitude were modulated by movement demands, being most pronounced during standing and diminishing with increased motor complexity. Auditory cue conditions showed independent effects of motor and cognitive demands without interaction, suggesting different neural processing strategies across sensory modalities.

## 4 Discussion

Our study illustrates how cognitive‐motor interference is modulated by cue modality and locomotion demands. We demonstrate that visual and auditory cueing elicit distinct cognitive control strategies: visual cues facilitated proactive motor preparation (evidenced by CNV amplitude increases in mixed‐task blocks), while auditory cues showed limited evidence of preparatory modulation but demonstrated reactive resource allocation (reflected in P3 amplitude patterns during target processing). Contrary to predictions from multiple resource theory and our Hypothesis 2, visual cues enhanced performance in complex locomotor conditions, with modality‐specific movement effects—perturbed walking reduced response times in auditory‐cued mixed‐task blocks but spared performance in visually‐cued conditions. These findings challenge assumptions about uniform cross‐modality interference and highlight the importance of cue presentation strategies in dynamic environments.

### 4.1 Cue Modality and Task‐Switching

Consistent with task‐switching literature, mixed‐task blocks elicited prolonged response times, reduced accuracy, and elevated subjective workload compared to repeat‐only conditions (Rubin and Meiran 2005). Supporting Hypothesis 1, perceived workload increased with both motor complexity (standing < walking < perturbation) and cognitive demands (repeat‐only < mixed‐task blocks). However, our prediction of modality‐specific workload differences was not supported—participants rated visual and auditory conditions similarly across all task combinations, suggesting that subjective workload reflects general dual‐task demands rather than modality‐specific resource competition.

Regarding behavioral performance (Hypothesis 2), cue modality did not impact subjective ratings but modulated behavioral outcomes: auditory cues decreased response times in repeat‐only blocks, whereas visual cues decreased response times in mixed‐task blocks. We also found behavioral switch costs similar to those in regular lab experiments (LaPlume et al. 2022). Contrary to our prediction that visual cues would impair performance in simpler tasks, visual cues showed advantages in complex (mixed‐task) rather than simple (repeat‐only) conditions. As predicted, we observed main effects of motor and cognitive difficulty on response accuracy as well, though the modality‐specific interference patterns were more nuanced than anticipated.

These patterns found in behavioral responses align with neural indices of cognitive control—visual cues elicited proactive preparation (elevated frontal/central CNV amplitudes in mixed‐task blocks), while auditory cues showed no significant task difficulty effects on preparatory activity but demonstrated reactive compensation (increased P3 amplitudes during target processing).

Testing Hypothesis 4, we found partial support for our predictions. Notably, CNV amplitude differences between switch/repeat trials did not reach statistical significance in either modality, contradicting our prediction of preparatory enhancement with cognitive demands in both modalities. However, visual cues showed a pronounced CNV increase from repeat‐only to mixed‐task blocks, suggesting preparatory resource allocation for anticipated task demands. This proactive strategy may explain the mixed‐task response time (RT) advantage for visual cues, as pre‐activated motor sets could expedite response execution. In contrast, auditory cue processing showed no significant preparatory modulation (CNV) but appeared to prioritize reactive control, with P3 amplitude increases in more demanding task conditions reflecting compensatory effort under high cognitive load. These modality‐specific strategies mirror the proactive/reactive framework of cognitive control outlined by Braver (2012).

### 4.2 Cue Modality and Cognitive‐Motor Interference

Prior literature postulates that locomotion can impair concurrent cognitive task performance (Bloem et al. 2001; Lajoie et al. 1993). In our experiment, we found that subjects rated walking, with balancing requirements, to be subjectively more demanding than standing and regular treadmill walking. This pattern is also reflected in gait parameters as subjects took shorter steps, therefore increasing their gait stability. Not supporting Hypothesis 3, gait analysis showed longer stride lengths during perturbation conditions, reflecting maladaptive postural control. Nonetheless, we found a decrease in stride length in mixed‐task blocks, therefore highlighting the effect of cognitive‐motor interference in this paradigm—as predicted, mixed‐task blocks showed gait modifications, with participants adopting more conservative stepping patterns to accommodate cognitive‐motor interference.

Interestingly, the motor × cognitive difficulty interaction for response times revealed that movement complexity effects were specific to task context. While repeat‐only blocks showed no movement‐related RT differences, perturbation conditions paradoxically improved response times during mixed‐task blocks. This dissociation suggests visual cues supported stable dual‐task performance by frontloading cognitive effort during preparation (CNV effects), whereas auditory cue processing competed with locomotor adaptation for reactive resources.

Examining our ERP hypotheses, the results provided partial support for our predictions. For Hypothesis 4, movement effects were confirmed in both modalities (auditory: standing > walking; visual: walking < perturbation). Auditory CNV showed heightened preparatory activity only during standing, while visual CNV was strongest during perturbed walking, contributing to observed reactive and proactive CMI effects.

Testing Hypothesis 5, P3 results partially supported our predictions. As hypothesized, P3 amplitudes diminished with motor load in both modalities (standing > walking > perturbation). However, the predicted amplification with cognitive complexity showed unexpected modality differences. In auditory conditions, P3 increased with cognitive demands as predicted (switch > repeat > repeat‐only), suggesting reactive resource mobilization. Surprisingly, visual conditions showed the opposite pattern (repeat‐only > mixed trials), and critically, a significant motor × cognitive difficulty interaction emerged. This interaction revealed that cognitive difficulty effects on P3 were present during standing but disappeared with movement, contradicting our prediction that auditory cues would better mitigate motor‐related P3 decreases. Instead, the interaction in visual conditions suggests that when movement demands increase, visual cueing elicits a different strategy—potentially leading to a pro‐active preparation of visual target processing in favor of maintaining stable motor‐visual coordination.

The convergence of neural and biomechanical metrics suggests that participants strategically reallocated resources from cognitive preparation to postural control when task complexity exceeded capacity limits.

### 4.3 Limitations

There are several limitations that need to be considered, though. First, treadmill walking lacks ecological stepping demands and environmental complexity, potentially underestimating real‐world cognitive‐motor tradeoffs. Second, exclusive use of visual targets precludes generalization to auditory‐target scenarios—future work should manipulate target modality to assess cue‐target congruency effects. Third, our workplace simulation, while controlled, omitted contextual stressors like time pressure or environmental distractions. Finally, the non‐significant CNV switch/repeat differences caution against overinterpreting preparatory neural markers without corroborating behavioral effects.

## 5 Conclusion

Our study demonstrates that cognitive‐motor interference patterns are fundamentally shaped by cue modality, with distinct proactive (visual) and reactive (auditory) control strategies governing performance. While our hypothesis testing revealed that basic dual‐task interference predictions were largely confirmed, the specific modality‐dependent patterns were more complex than anticipated. Contrary to predictions from Wickens' (2002, 2008) multiple resource theory, visual cues enhanced dual‐task efficiency during perturbed locomotion through anticipatory motor preparation—indicated by increased CNV amplitudes in mixed‐task blocks and reduced P3 demands during target processing. This proactive mechanism allowed visual‐cued performance to remain stable across movement conditions, challenging assumptions about uniform cross‐modality interference. Auditory cues, while advantageous in repeat‐only standing conditions, showed movement‐dependent preparatory activity and reactive resource allocation patterns in more demanding scenarios, as reflected in locomotor‐dependent RT fluctuations and task difficulty‐related P3 modulation. This pattern in auditory cue conditions might be due to transformational processes from auditory to visual information processing, as suggested by Baddeley et al.'s (2001) working memory model, culminating in patterns common in CMI literature—increasing motor task demands impairing cognitive resource availability (Debener et al. 2012; Reiser et al. 2019, 2021, 2022; Shaw et al. 2018).

Critically, these modality‐specific effects emerged despite non‐significant CNV differences between switch/repeat trials, redirecting focus to preparation differences between single/mixed‐task blocks rather than trial‐level switching. The dissociation between preserved visual‐cued performance and locomotion‐sensitive auditory‐cued responses underscores the importance of cue presentation systems in workplace design: HMD‐based visual cues appear optimal for complex mobile tasks, while auditory interfaces may suffice for low‐demand scenarios.

The most striking finding was the reversal of expected modality‐specific disadvantages: visual cues showed stability and advantages in complex conditions rather than the predicted visuospatial competition costs, while auditory cues demonstrated reactive rather than proactive control strategies.

Overall, the study results showed that cognitive‐motor interference as seen in previous investigations might not entirely be due to locomotor effort, but rather the overall walking stability demands that originate in the imperfection of the ground surface and feature‐richness of the environment (Matthis et al. 2018; Wascher et al. 2022). Nonetheless, we found significant and fundamental differences in how information from commonly used mobile presentation devices is processed and how they impact performance.

These findings refine theoretical frameworks by showing how Braver's (2012) proactive/reactive control continuum interacts with sensory modality under mobility constraints. For applied contexts, they highlight that cognitive‐motor interference arises not merely from locomotor effort, but from competition between task‐specific control strategies—a critical consideration for designing assistive technologies in dynamic workplaces.

## Author Contributions

**Julian Elias Reiser:** conceptualization, methodology, data curation, formal analysis, investigation, project administration, visualization, writing – original draft, writing – review and editing, resources. **Gerhard Rinkenauer:** conceptualization, writing – review and editing, resources. **Stefan Arnau:** writing – review and editing, resources. **Lewis L. Chuang:** conceptualization, writing – review and editing. **Edmund Wascher:** writing – review and editing, conceptualization, resources.

## Conflicts of Interest

The authors declare no conflicts of interest.

## Supporting information


**Data S1:** psyp70122‐sup‐0001‐DataS1.zip.

## Data Availability

The anonymized EEG datasets, quantified gait measure matrices, and NASA‐TLX scores that were used in this manuscript are publicly available in this Zenodo repository: https://doi.org/10.5281/zenodo.14506178. The used MATLAB scripts are available in a public GitHub repository: https://github.com/julianreiser/grail_switch.git.
