# Supplementary material for: How Do Humans Process Audiovisual Cues for Task‐Switching While Walking? An EEG/ERP Study
Source: Psychophysiology. 2025 Aug 4;62(8):e70122. doi: 10.1111/psyp.70122 (PMC12319377; doi:10.1111/psyp.70122)

# FMUA Cluster means

## CNV auditory motor difficulty

##
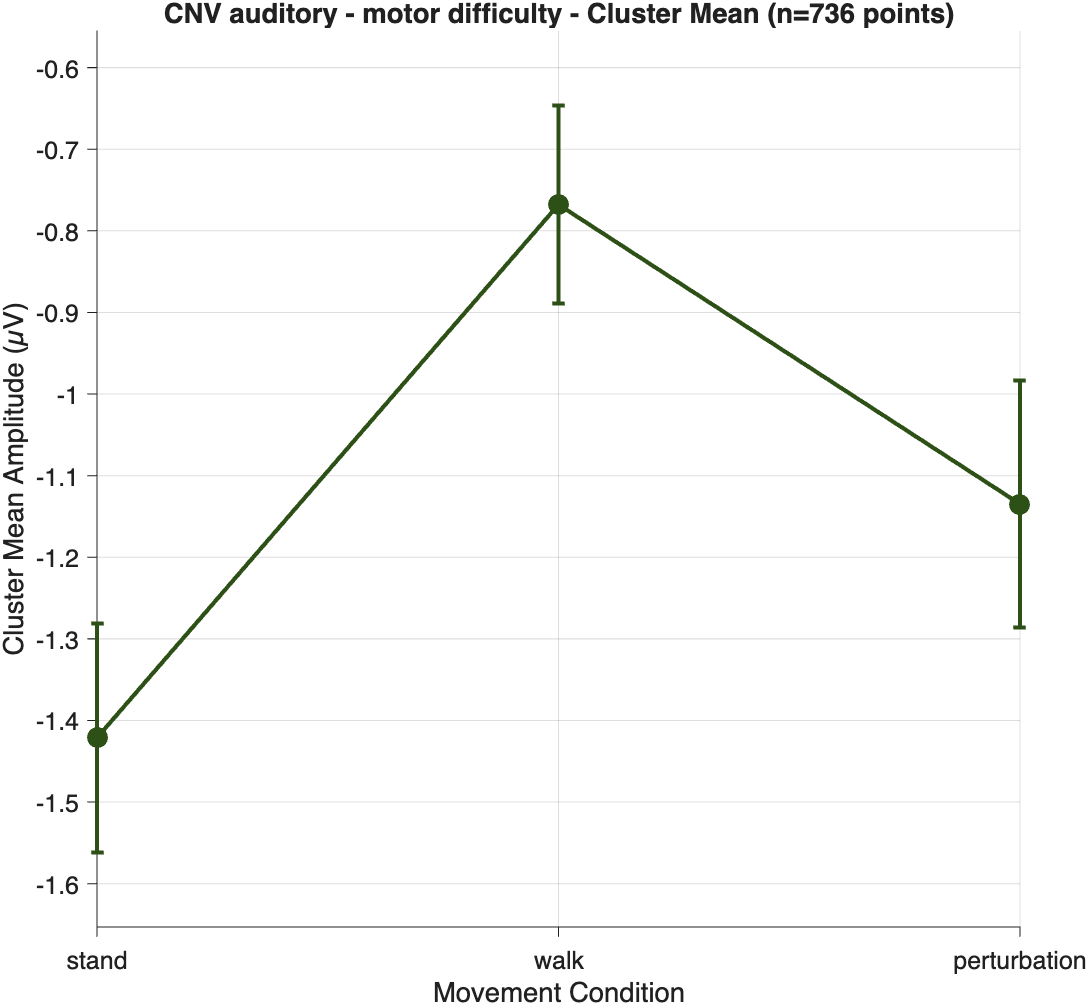


## CNV auditory cognitive difficulty


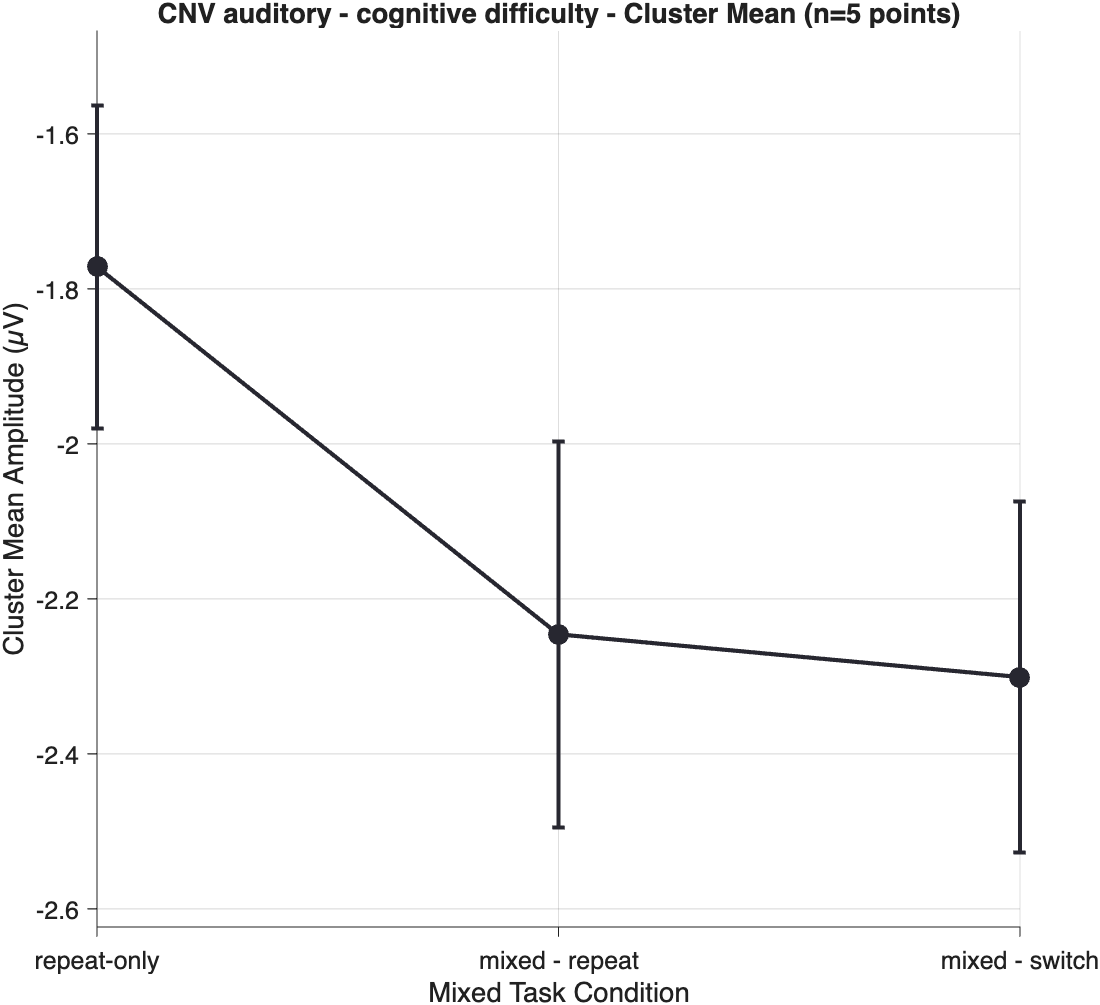


## CNV visual cognitive x motor difficulty


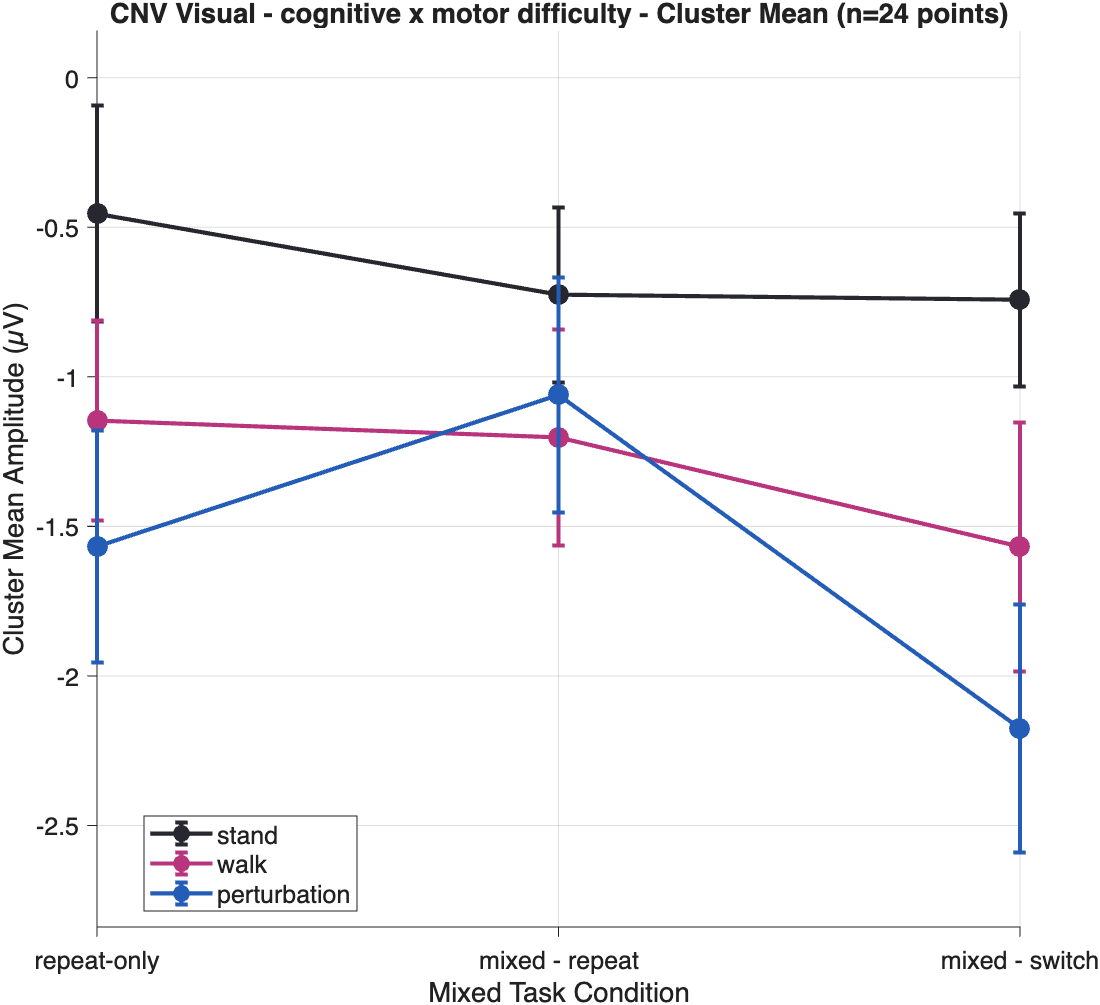


## CNV visual motor difficulty


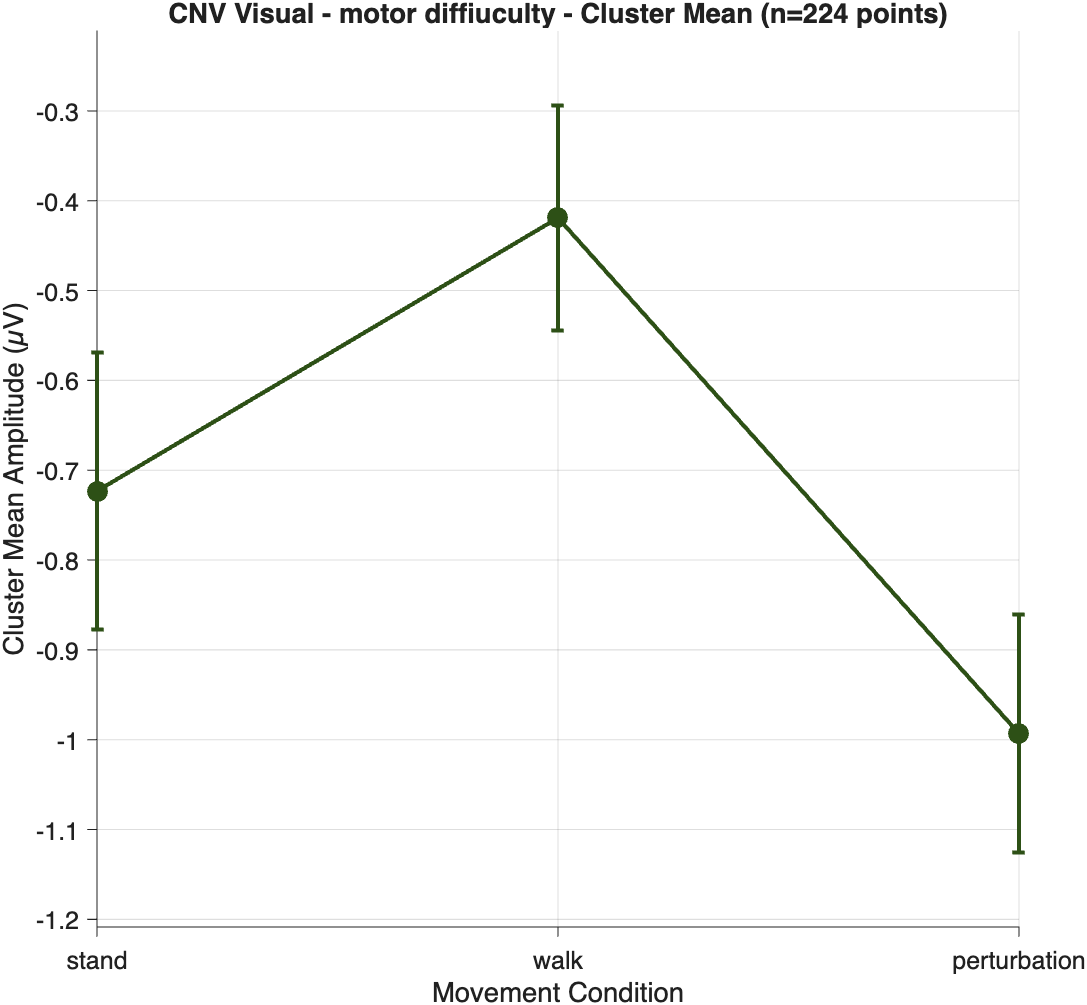


## CNV visual cognitive difficulty


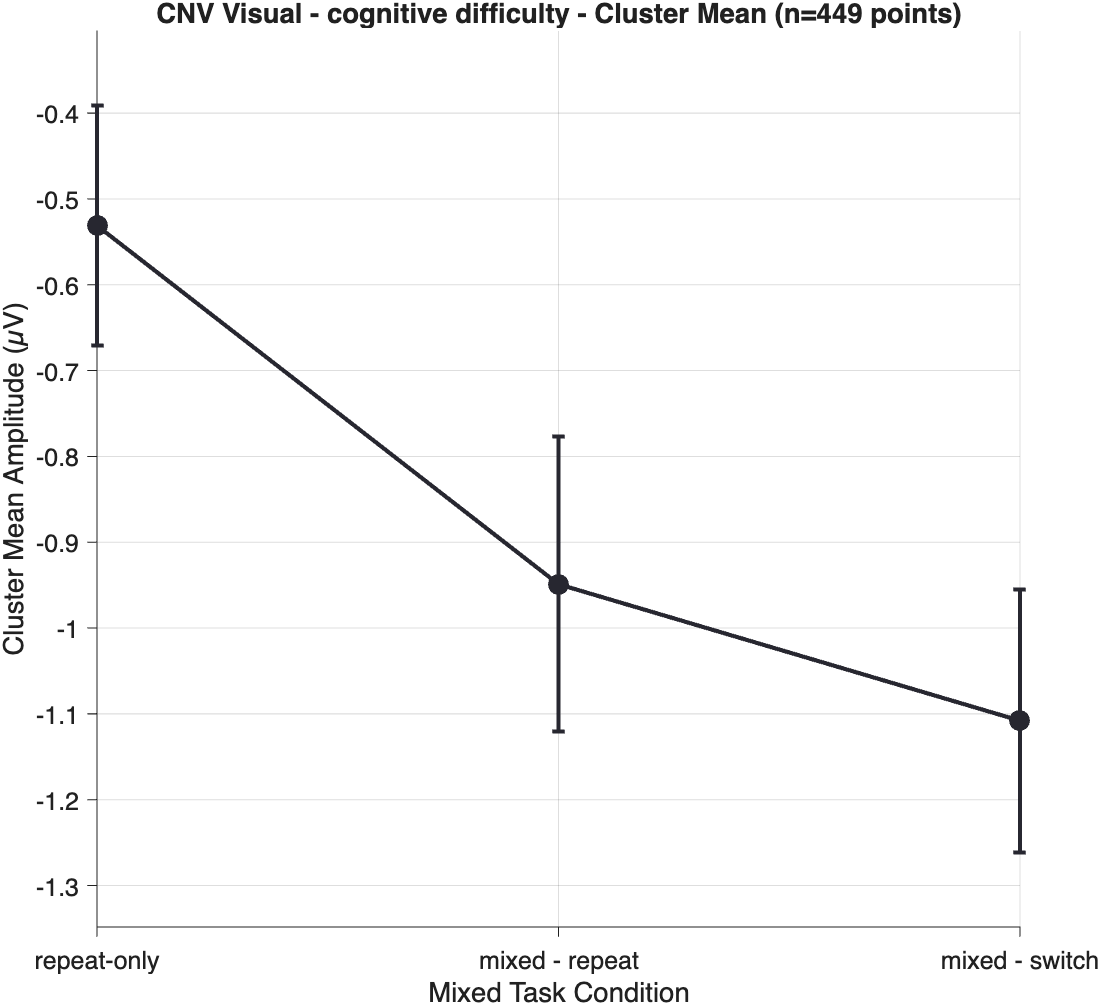


## P3 auditory cognitive x motor difficulty

##
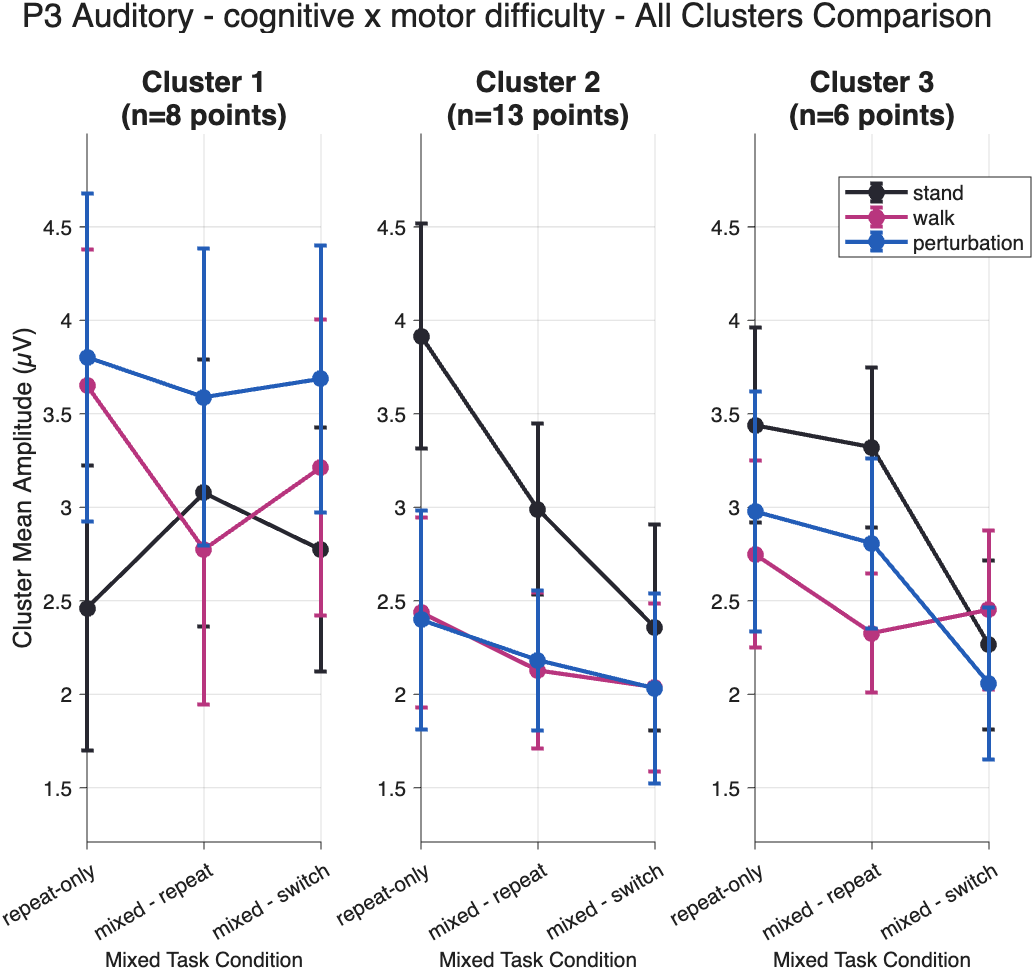


## P3 auditory motor difficulty


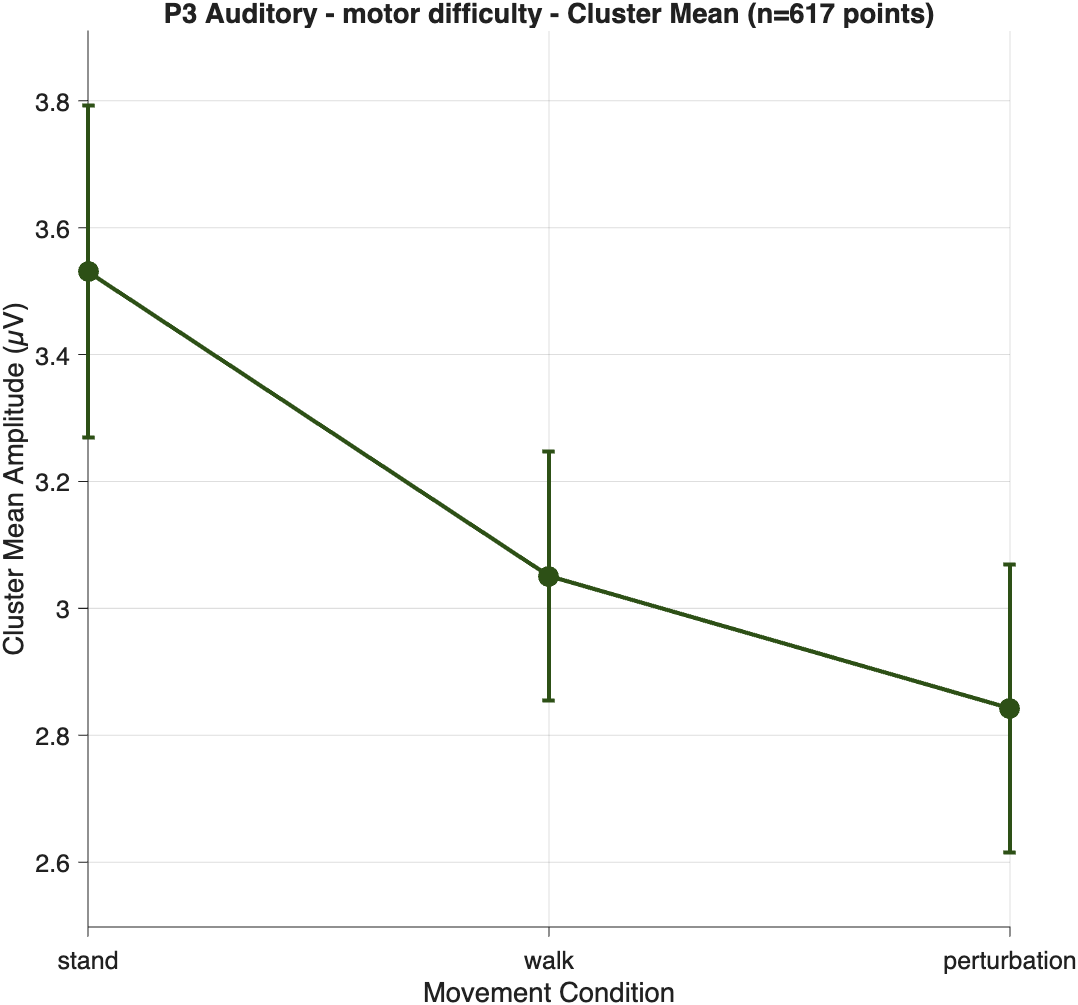


## P3 auditory cognitive difficulty


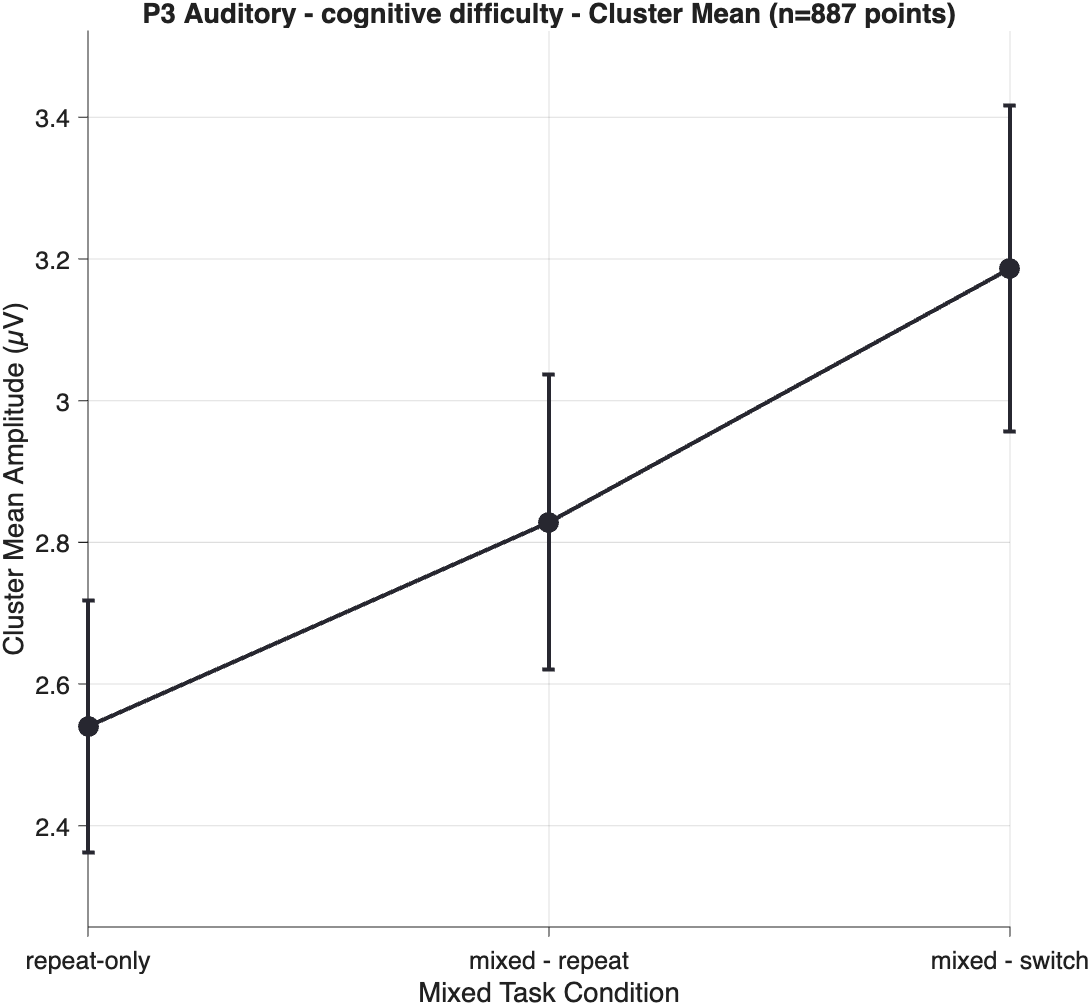


## P3 visual cognitive x motor difficulty


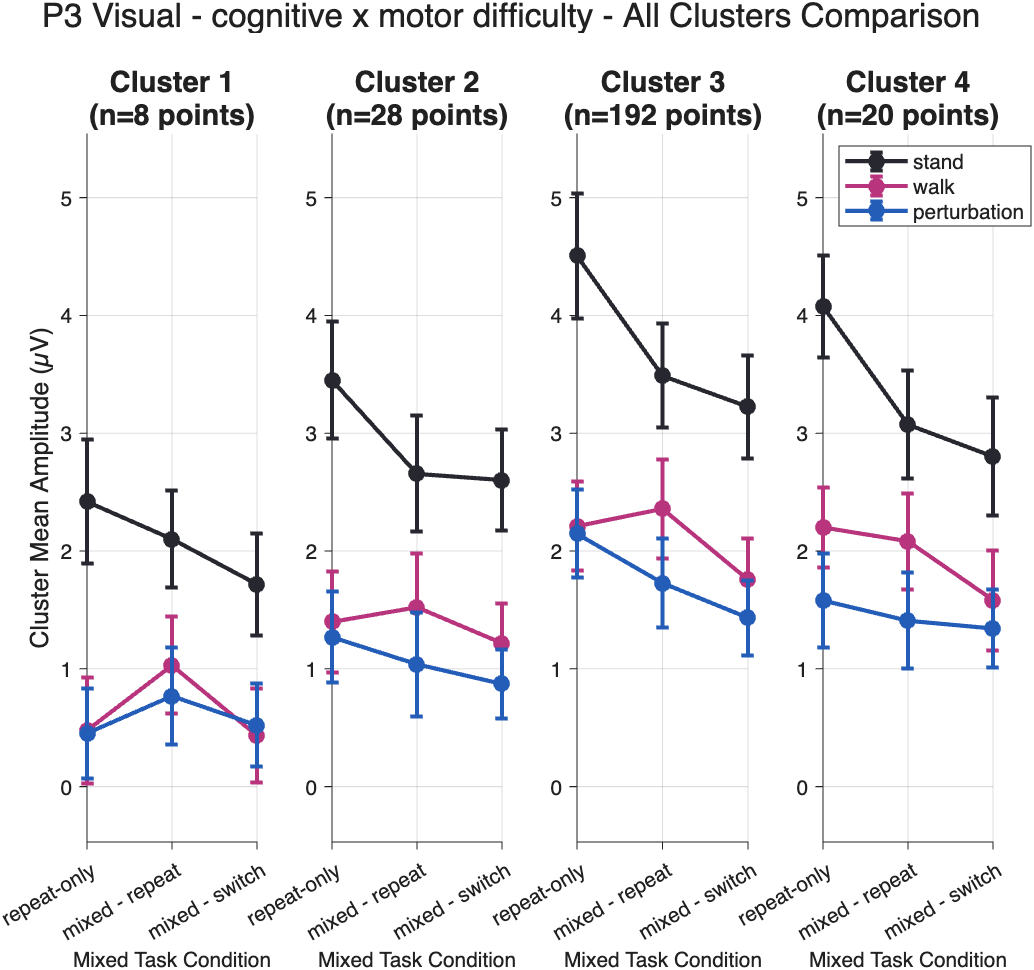


## P3 visual motor difficulty


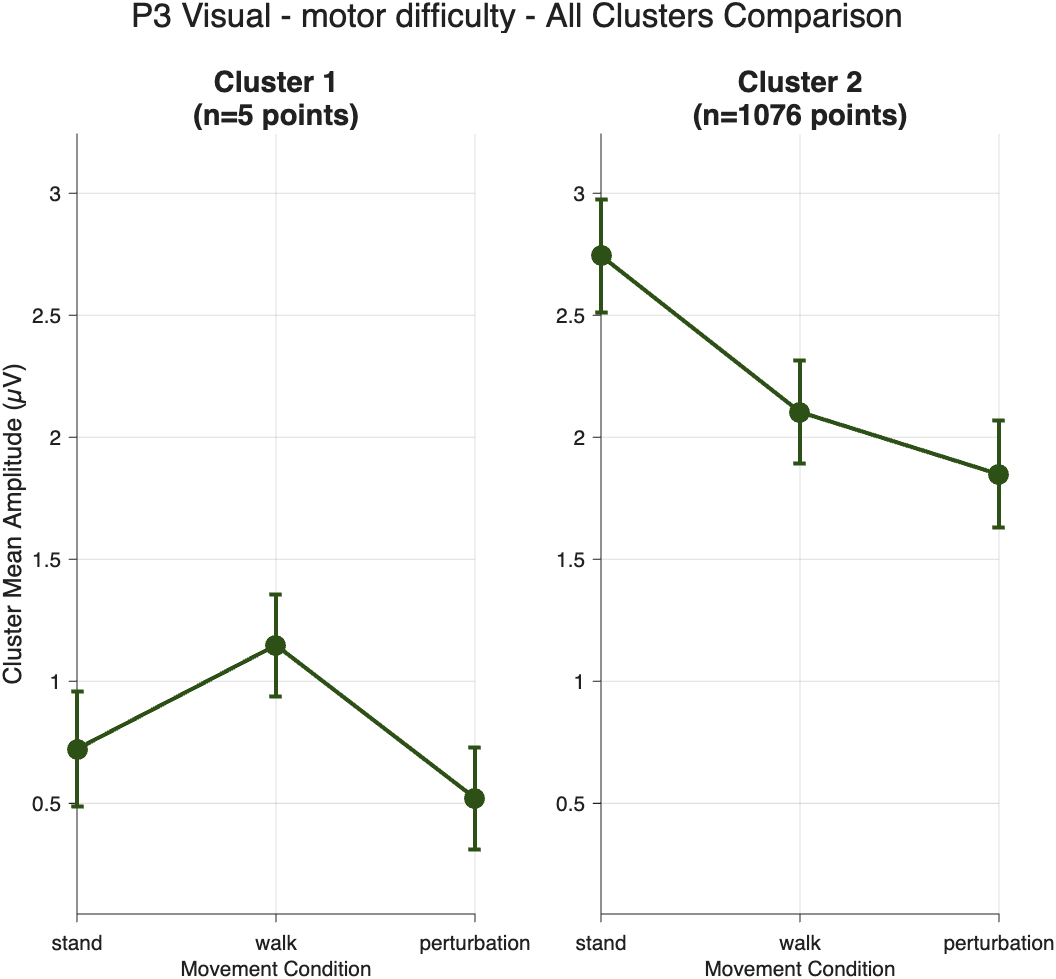


## P3 visual cognitive difficulty


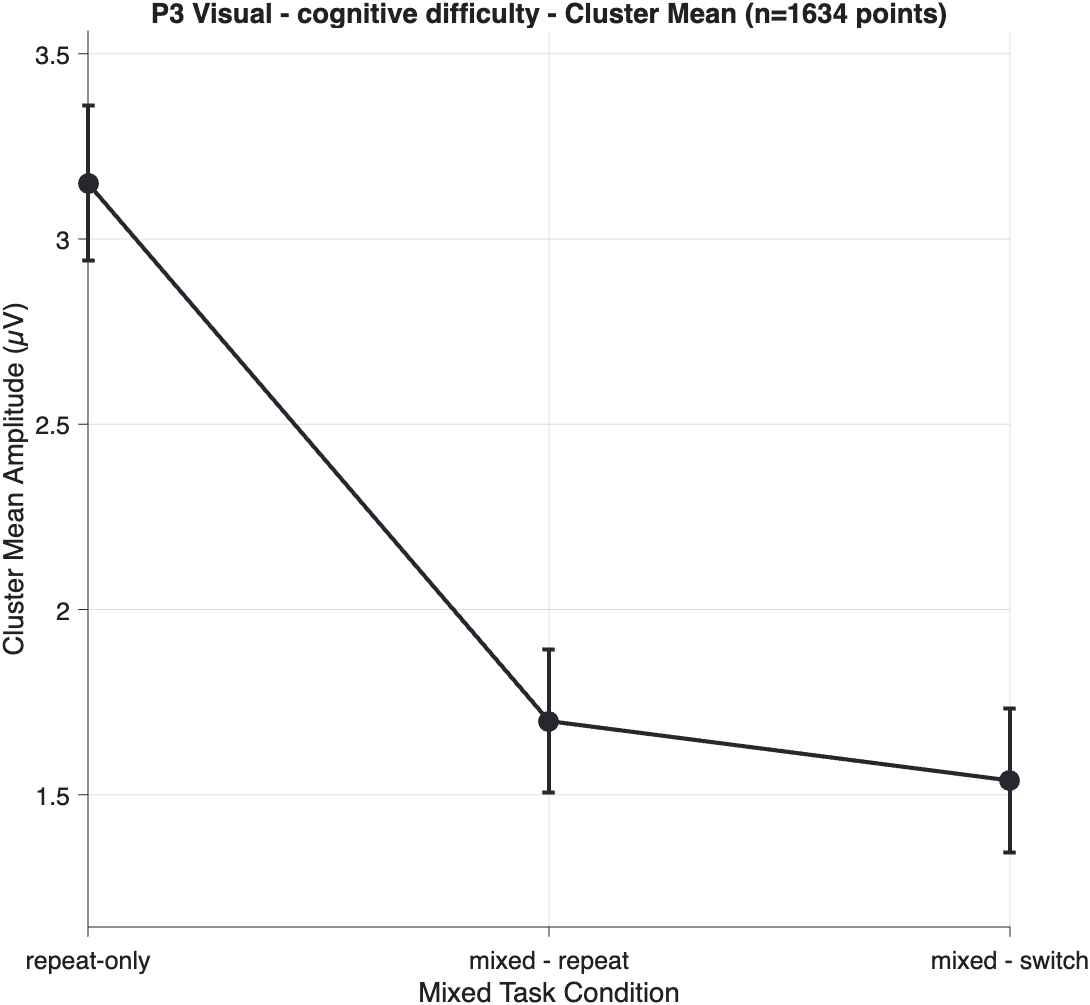


# FMUA Cluster effect sizes (partial eta squared)

## CNV auditory motor difficulty


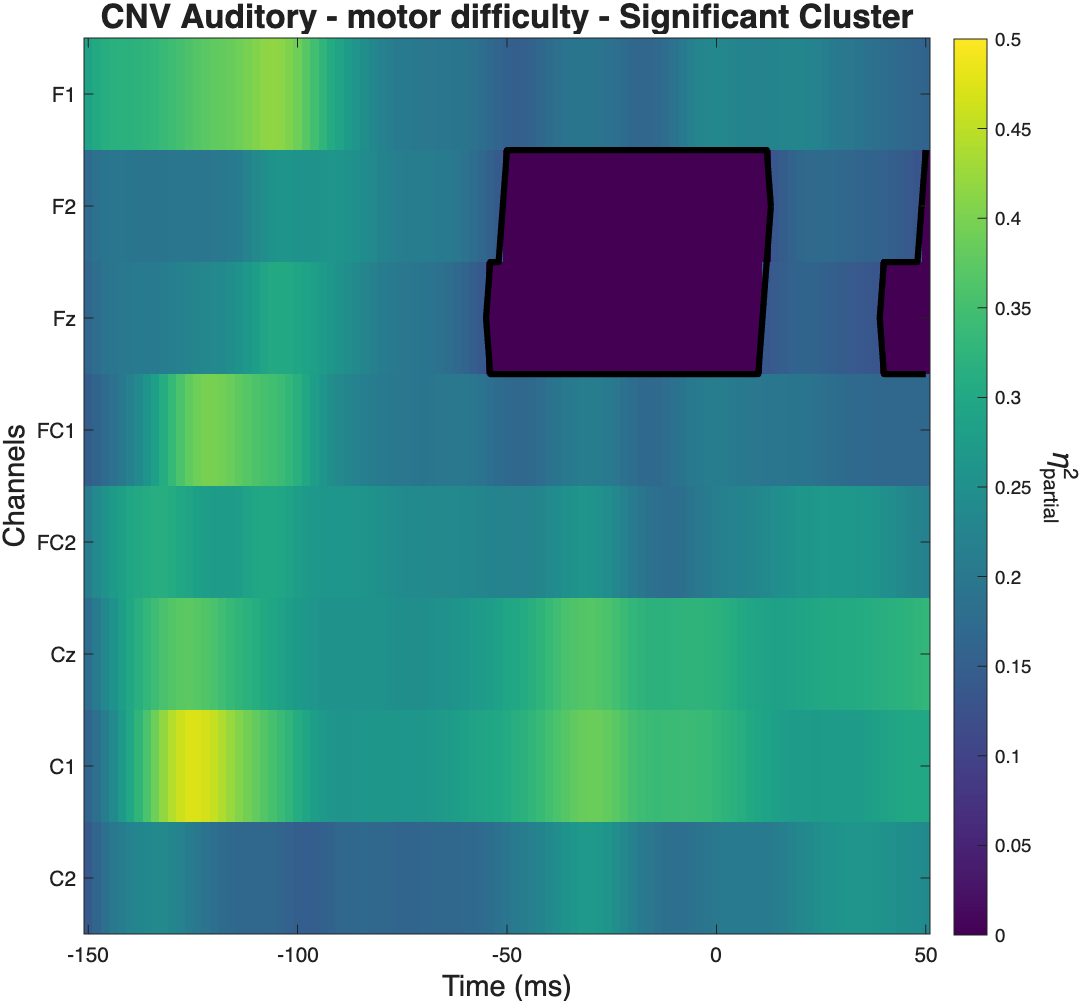


## CNV auditory cognitive difficulty


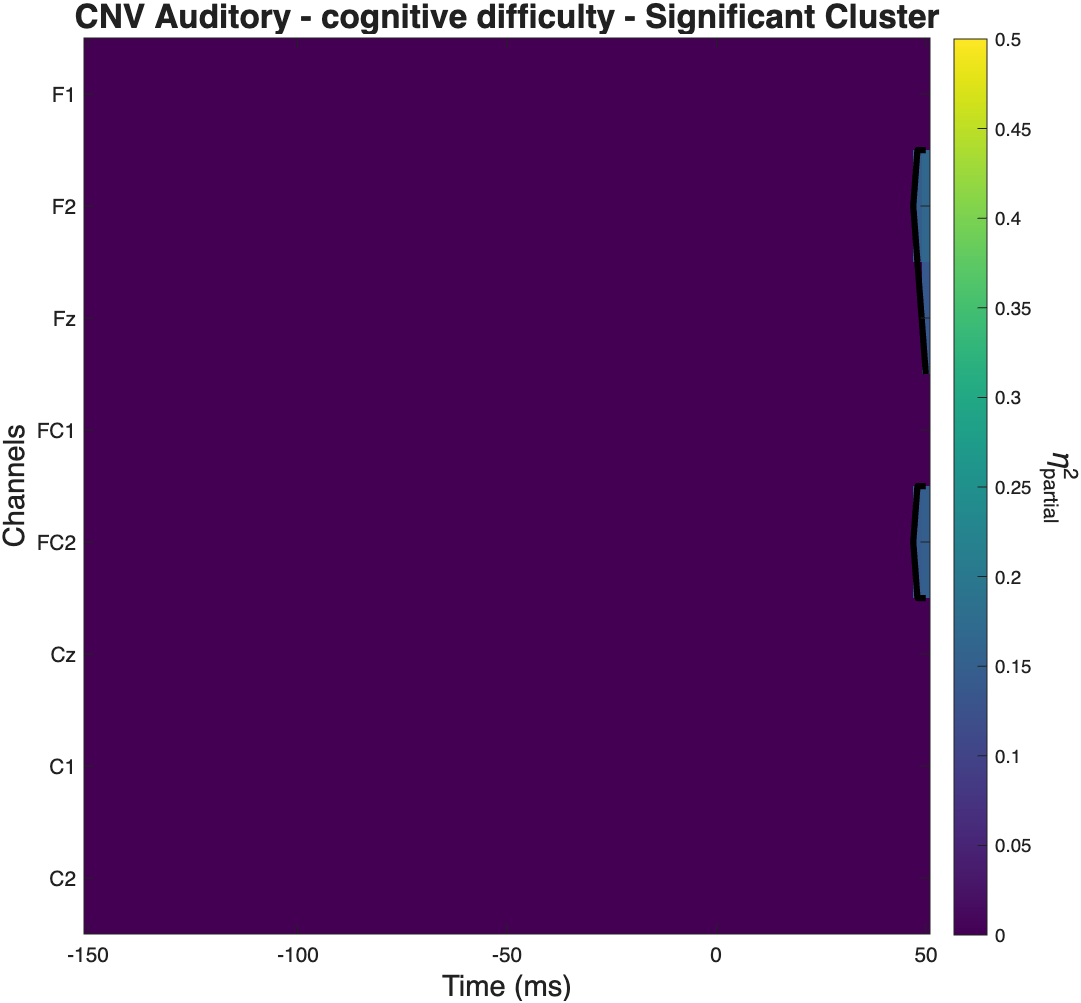


## CNV visual cognitive x motor difficulty


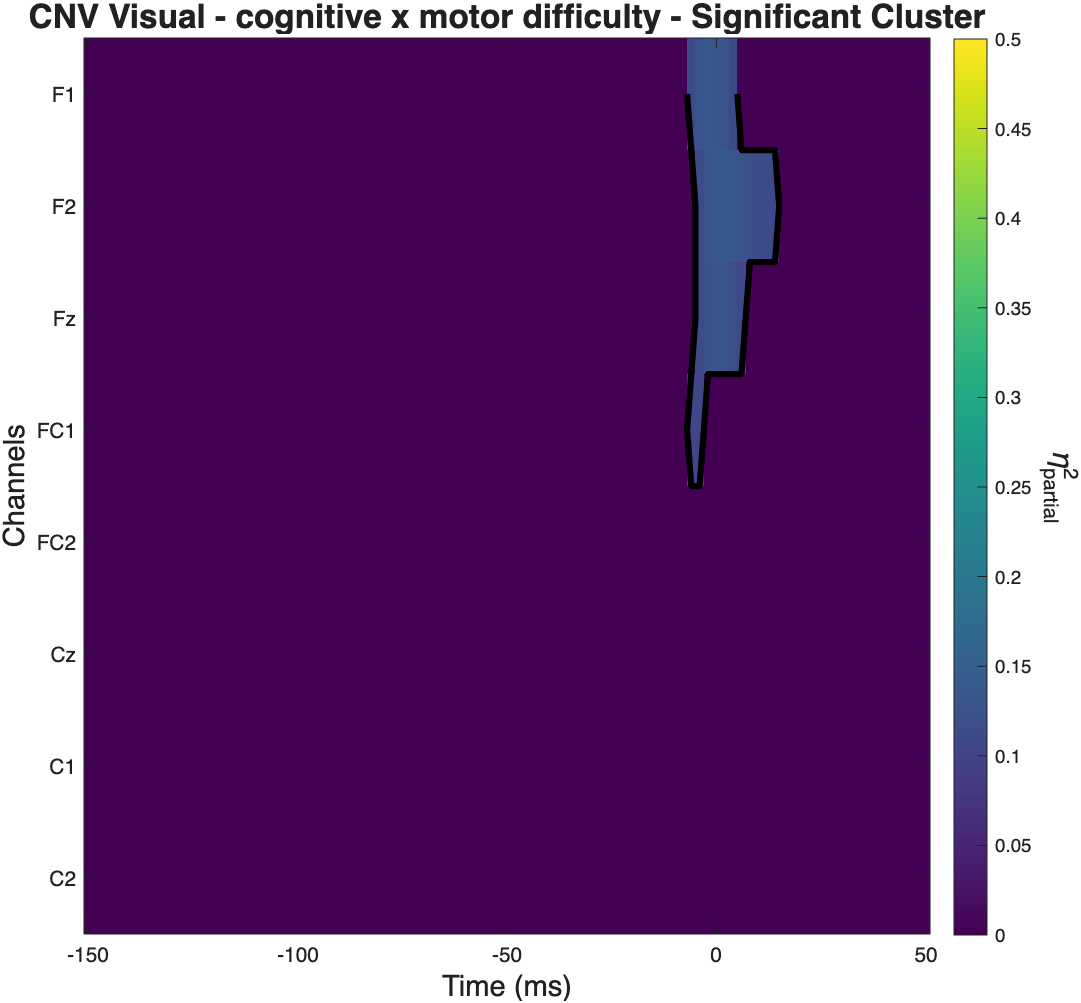


## CNV visual motor difficulty


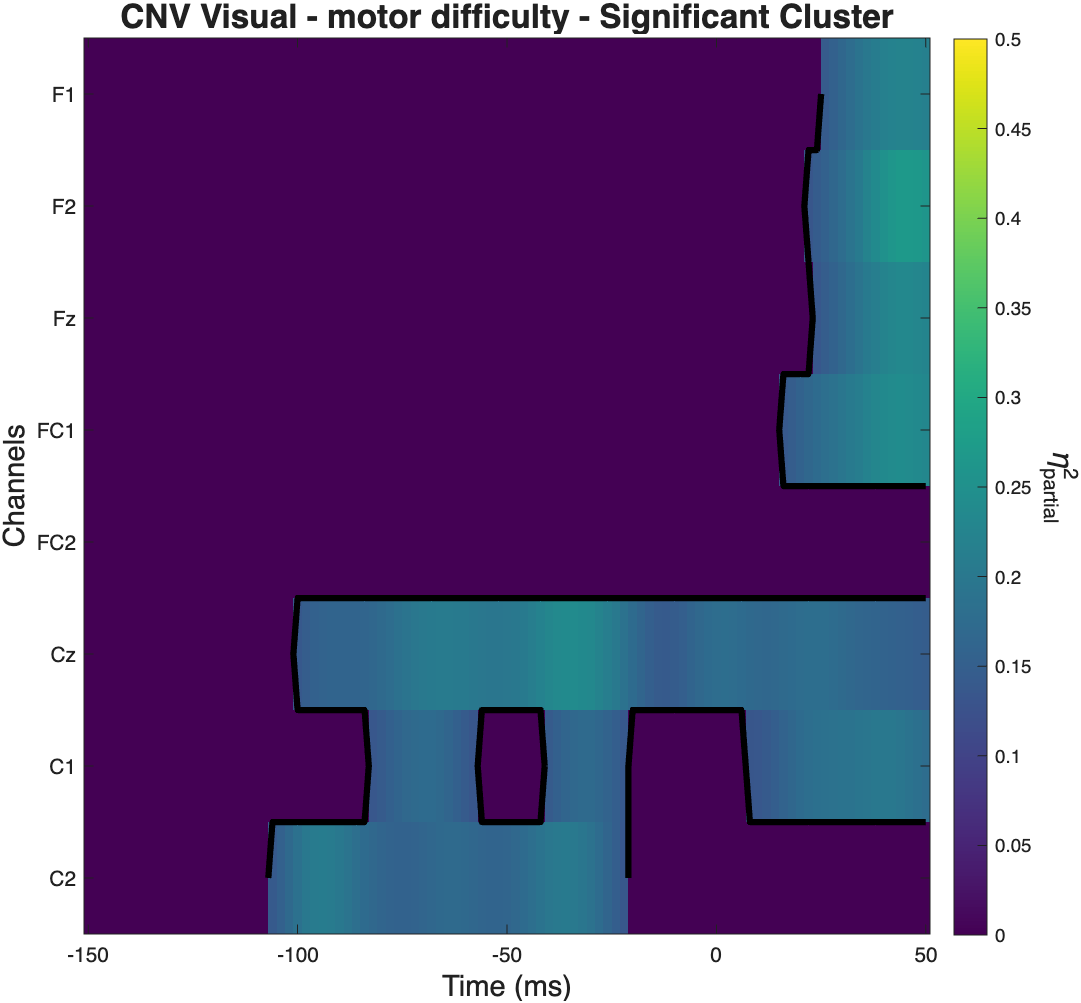


## CNV visual cognitive difficulty


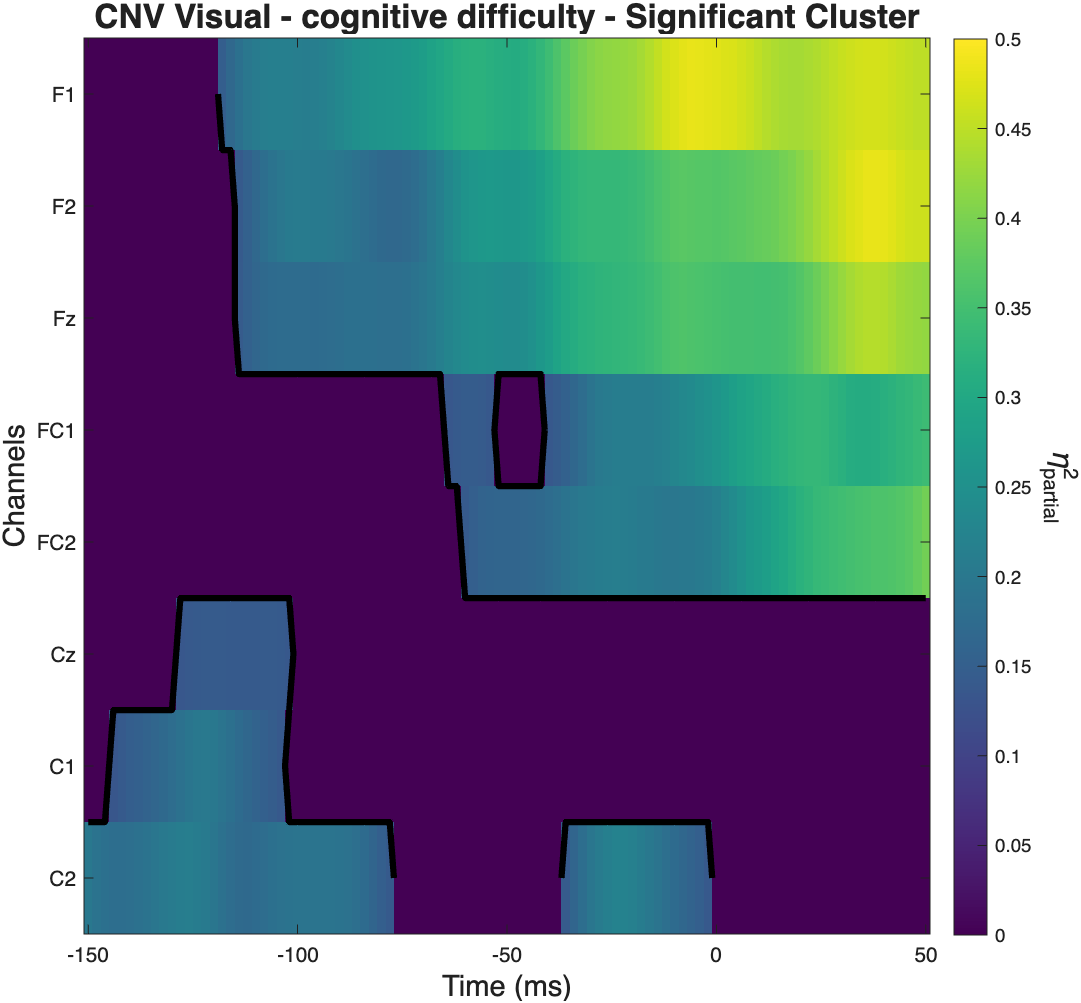


## P3 auditory cognitive x motor difficulty


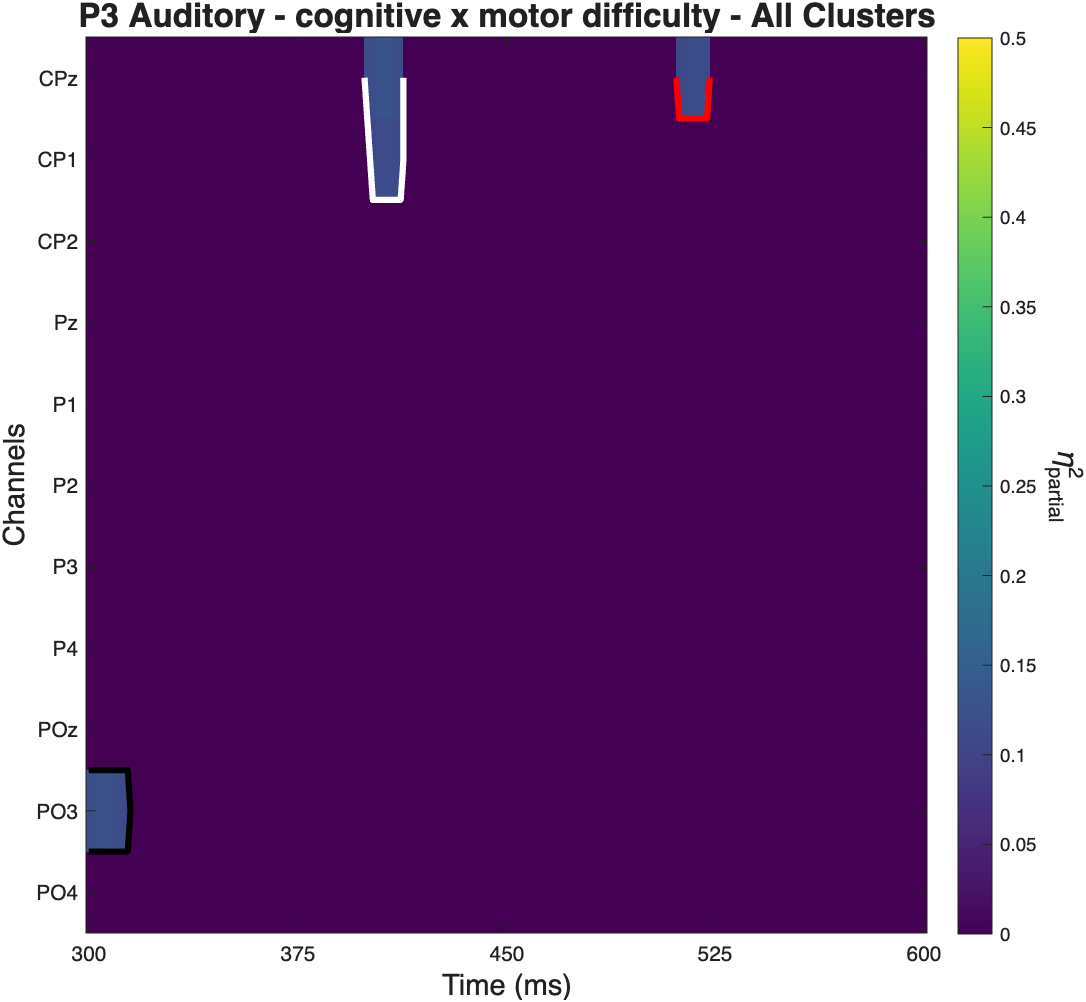


## P3 auditory motor difficulty


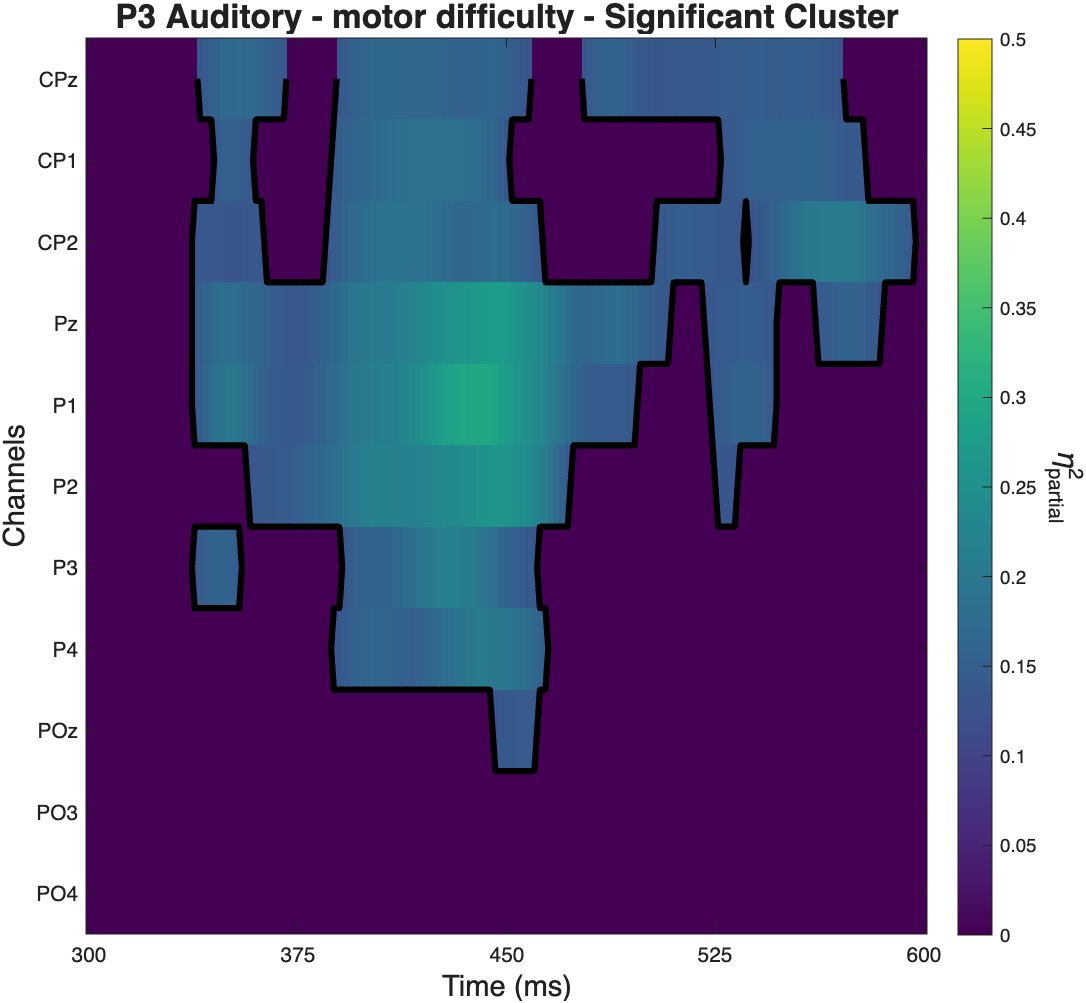


## P3 auditory cognitive difficulty


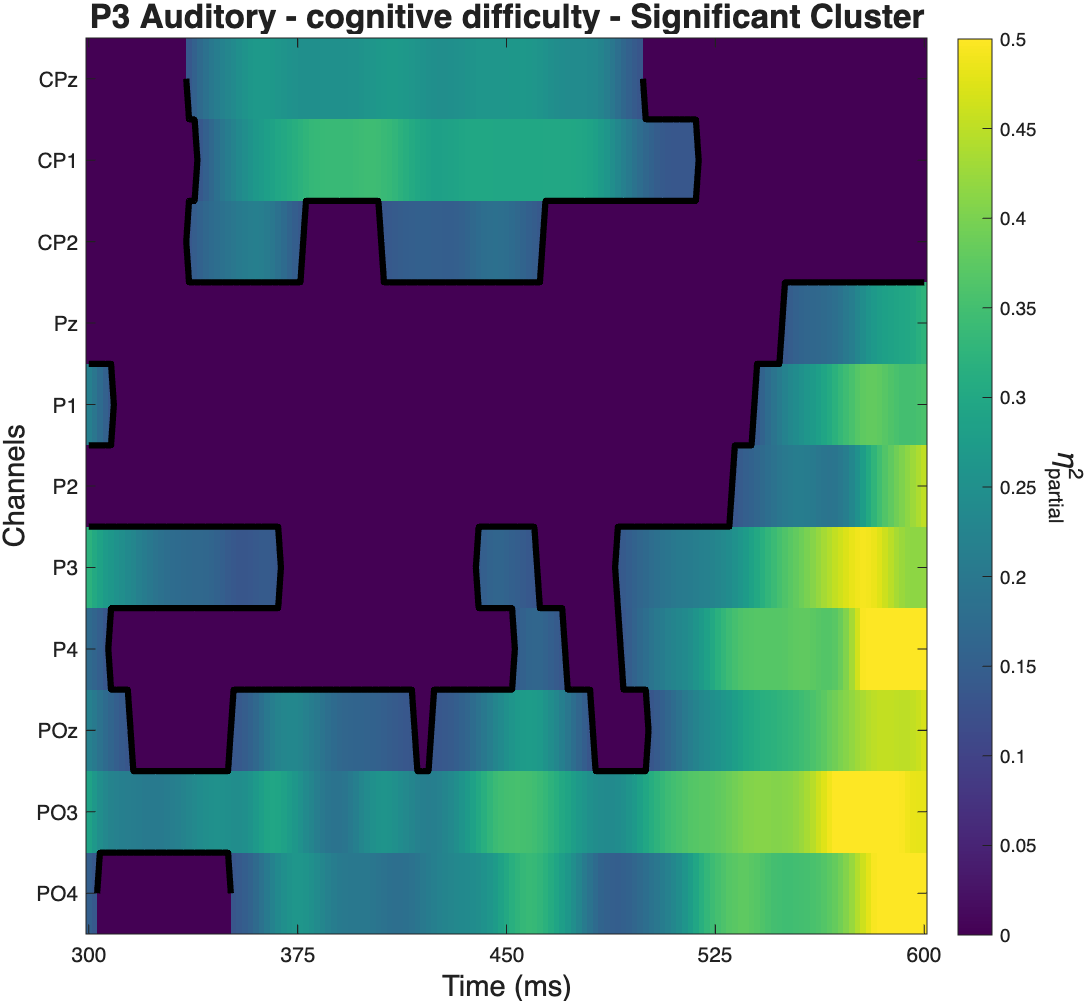


## P3 visual cognitive x motor difficulty


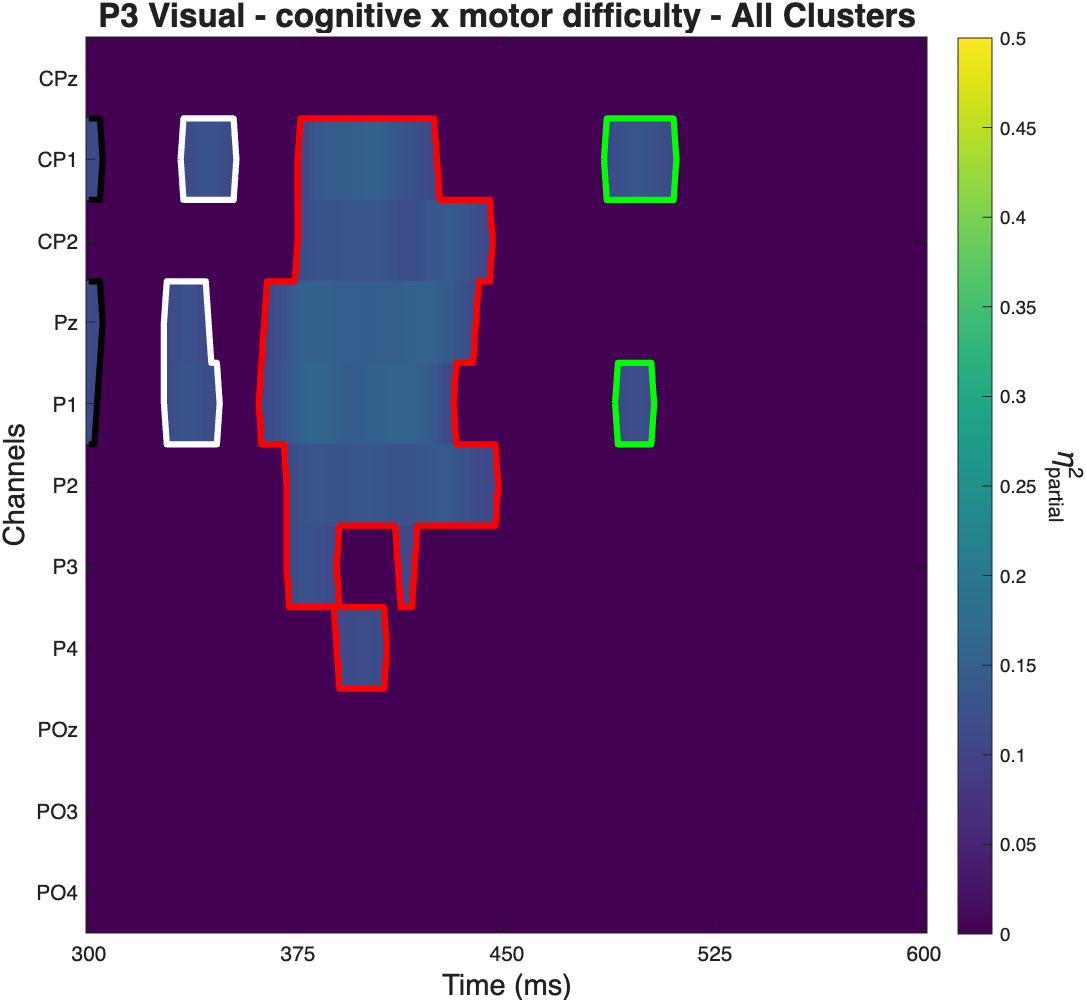


## P3 visual motor difficulty


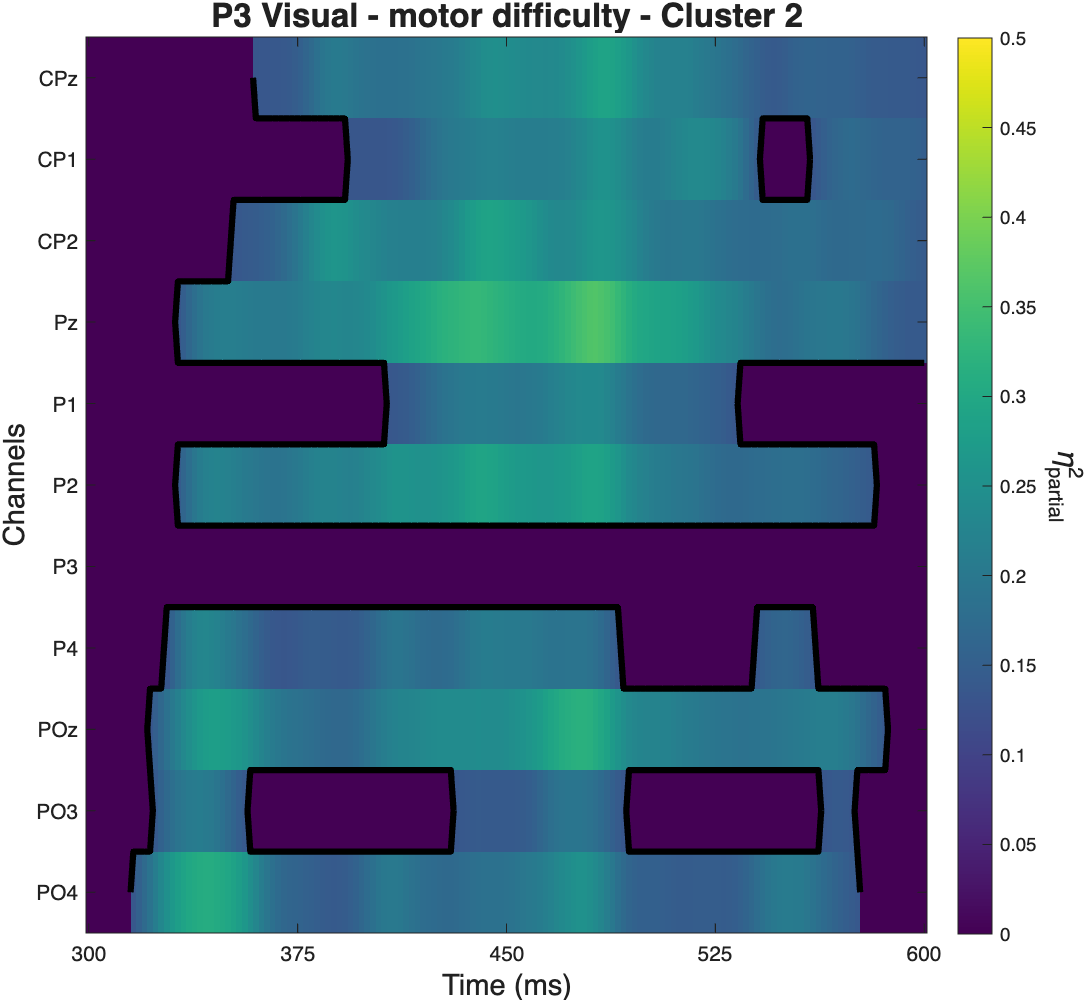


## P3 visual cognitive difficulty


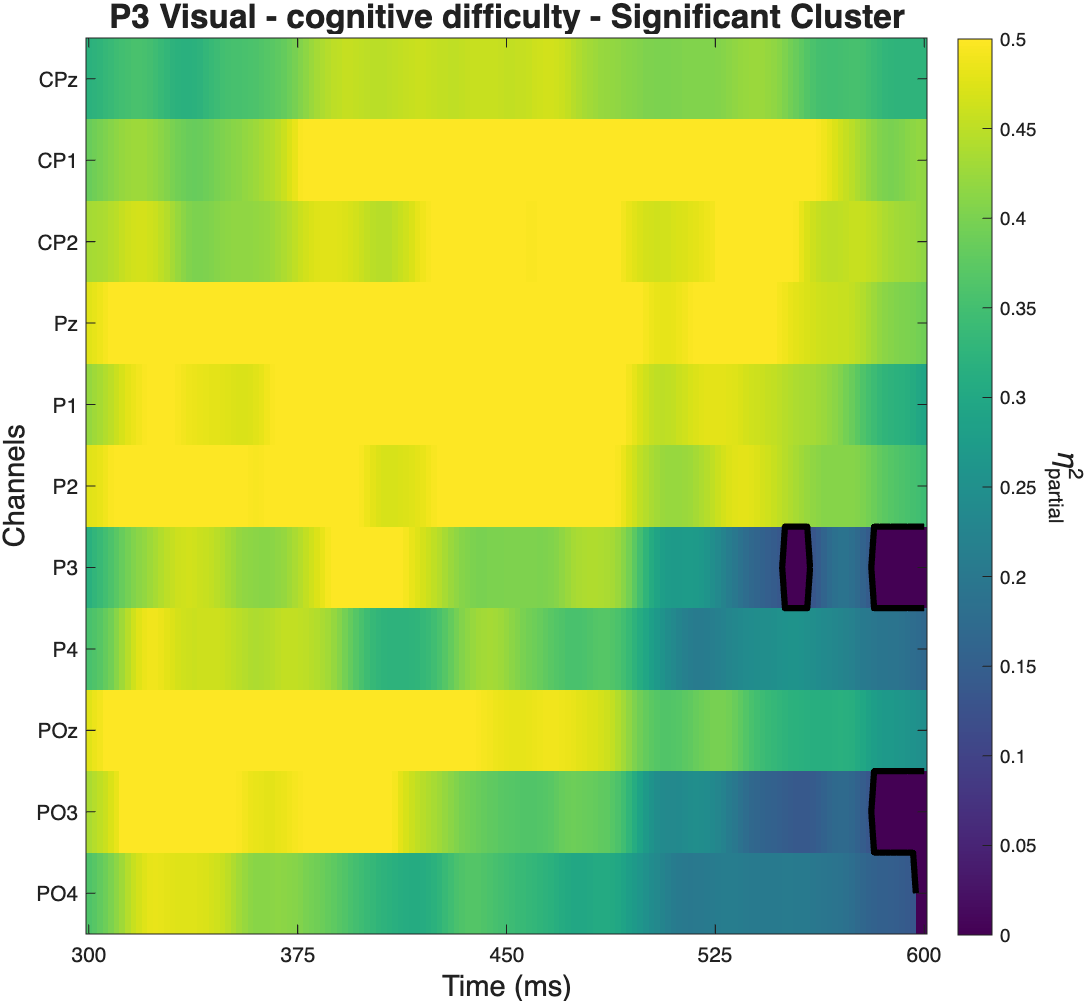

Supplement: Supplementary file 1 — Data S1: psyp70122‐sup‐0001‐DataS1.zip. [file PSYP-62-e70122-s001.zip › Reiser_Psychophysiology_SupplementaryMaterials_Revision.docx]
